# Changes in the Nationwide Incidence of Bell's Palsy in the General Population Before and After the COVID‐19 Pandemic: An Ecological Study

**DOI:** 10.1002/jmv.70431

**Published:** 2025-06-02

**Authors:** Seungyeon Lee, Nang Kyeong Lee, Seung Won Lee, Yong Joon Kim

**Affiliations:** ^1^ Department of Ophthalmology, Institute of Vision Research Yonsei University College of Medicine Seoul Republic of Korea; ^2^ Department of Precision Medicine, School of Medicine Sungkyunkwan University Suwon Republic of Korea

**Keywords:** Bell's palsy, coronavirus disease 2019, non‐pharmaceutical interventions, vaccination

## Abstract

This nationwide population‐based ecological study investigated changes in the incidence of Bell's palsy (BP) in South Korea during the COVID‐19 pandemic. Patients diagnosed with BP between January 2017 and December 2022 were analyzed. Considering the unique progression of the COVID‐19 pandemic and corresponding public health responses in South Korea, the study period was divided into four phases: pre‐COVID‐19 (2017–2019), non‐pharmaceutical intervention (NPI, 2020), nationwide vaccination (2021), and nationwide infection (2022). Poisson regression was used to calculate incidence rate ratios (IRRs) for each pandemic phase compared to the pre‐COVID‐19 baseline, adjusting for age and sex. Among 103 482 patients, BP incidence significantly decreased during the vaccination (IRR 0.96; 95% CI, 0.94–0.98) and infection (IRR 0.95; 95% CI, 0.93–0.97) phases (*p* < 0.001). These trends were more pronounced among women and older adults and did not correlate with national vaccination or infection rates. Our findings suggest that the incidence of BP did not increase during the COVID‐19 era at the level of the general population nationwide. Further studies using individual‐level data on COVID‐19 infection and vaccination are needed to clarify their potential direct impact on BP incidence.

## 1 Introduction

Severe acute respiratory syndrome coronavirus 2 (SARS‐CoV‐2), the virus that causes coronavirus disease (COVID‐19), triggered an acute respiratory disease outbreak in December 2019, continuously affecting populations worldwide. On March 11, 2020, the World Health Organization (WHO) declared COVID‐19 a global pandemic. Since then, approximately 776 million COVID‐19 cases and over 7 million COVID‐19‐related deaths have been reported globally as of October 2024 [1].

Nations worldwide responded to the pandemic by implementing strict non‐pharmaceutical interventions (NPIs), such as social distancing, mandatory face mask use, and asymptomatic testing to reduce the spread of infectious pathogens [2]. In December 2020, the first COVID‐19 vaccines were developed, and vaccination programs were initiated worldwide. To date, over 5.6 billion individuals worldwide have received at least one dose of a COVID‐19 vaccine [1].

With the progression of the COVID‐19 pandemic and an increase in cases, extrapulmonary complications, including neurologic complications like Bell's palsy (BP), are being reported. BP, the most common disease of the facial nerve, presents as acute, unilateral palsy of the facial nerve lower motor neuron [3]. Reactivation of viruses such as herpes simplex virus (HSV) at the geniculate ganglion of the facial nerve may be responsible for most cases [4]. Similarly, few studies have raised concerns about an increase in BP incidence following SARS‐CoV‐2 vaccination or infection [5, 6, 7].

During the COVID‐19 pandemic, South Korea exhibited several distinctive characteristics that made it a suitable setting for conducting population‐level ecological analyses of exposure to vaccination and infection (Supporting Information S1: Figure S1). From February 2020, the South Korean government adopted an active NPI, and only 0.15% of the total population was infected with SARS‐CoV‐2 until February 2021, positioning South Korea as one of the top nations controlling the spread. COVID‐19 vaccines became available in February 2021, and by the end of that year, approximately 90% of the total population and 98% of the adult population had received at least one dose [8]. The stability in infection rates after the vaccinations changed dramatically when the Omicron variant became dominant from January 2022 [9]. The infection rate sharply increased, resulting in nearly 50% of the population being infected by mid‐2022, with over 60% infected by the end of 2022. Given the near‐universal exposure to both vaccination and infection, it is inherently difficult to identify an appropriate unexposed control group. Therefore, we employed an ecological study design to assess the incidence of BP across four phases in South Korea—NPI (2020), vaccination (2021), and infection (2022)—and compared these findings with pre‐pandemic data (2017–2019).

## 2 Materials and Methods

### 2.1 Data Extraction and Study Design

This study used the Korean Health Insurance Review and Assessment (HIRA) claims data from 2014 to 2022. In South Korea, nearly 97% of the entire population is enrolled in the national health insurance service. The database records comprehensive details on patients, including demographic profiles, diagnostic codes, procedures, prescribed medications, and direct medical expenditures for both inpatient and outpatient services. The HIRA Deliberative Committee approved the conditional use of the deidentified data (approval number M20230828002).

The customized HIRA data set provided for individual researchers is limited to a total size of less than 300 GB and does not include information on COVID‐19 vaccination. Therefore, this study was designed as an ecological study to examine the population‐level impact of exposure to NPI, vaccination, and infection, taking into account the unique circumstances of the COVID‐19 pandemic in South Korea.

### 2.2 Study Population

This study included data on 342 608 patients diagnosed with BP (Korean Standard Classification of Disease [KCD]−8 code G51.0) between 2017 and 2022 (Supporting Information S1: Figure S2). The index date was defined as the date of the earliest BP claim, and the patient was considered an incident case in that year. To exclude previously diagnosed patients, a 3‐year washout period was implemented, excluding 28 724 patients with registered BP codes from January 1, 2014 to December 31, 2016. Subsequently, 160 023 patients who had been diagnosed at least once with disease codes related to conditions that could mimic BP or other possible causes of facial nerve injury were excluded.

A total of 150 861 patients were newly diagnosed with BP between 2017 and 2022. Among them, 103 482 patients were prescribed glucocorticoid treatment within 60 days of their BP diagnosis, and the data of these patients were included in the main analysis. Definitions of disease codes and drug treatments are provided in Supporting Information S1: Table S1. Although the cases of BP diagnoses include codes registered up to December 31, 2022, the follow‐up data for these patients were utilized until February 28, 2023.

### 2.3 Outcome Measurements

Data were categorized into four phases: pre‐COVID‐19 (2017–2019), NPI (2020), nationwide vaccination (2021), and nationwide infection (2022). The annual incidence of newly diagnosed BP was calculated for each year. The crude incidence rate was computed per 100 000 persons using mid‐year population data provided by the Korean statistical information service (Kosis.kr, accessed in September 2024, Supporting Information S1: Table S2). Due to the characteristics of the customized HIRA data set, only patients diagnosed with BP were extracted, and information on their source population was limited to age and sex, as provided by the Korean Statistical Information Service. Accordingly, the unadjusted incidence rate ratio (IRR) and the age‐ and sex‐adjusted IRRs were calculated for each year and compared to those of 2019. Subgroup analyses were conducted by dividing the study population into males and females and according to four age groups ( ≤ 19, 20–39, 40–64, and 65–99 years).

### 2.4 Sensitivity Analysis

A sensitivity analysis was performed to enhance the validity of the outcome. When BP was defined using the KCD‐8 code combined with glucocorticoid prescriptions, mild cases not requiring treatment may have been excluded from the analysis. To address this, an alternate definition for BP, which defined incident BP solely based on the KCD‐8 code without requiring glucocorticoid prescription claims, was adopted in the sensitivity analysis. Baseline characteristics, incidence of BP, and age‐ and sex‐adjusted IRRs were calculated for each year and compared to those of 2019, similar to that for the main study population.

### 2.5 Statistical Analysis

The unadjusted and adjusted IRRs were calculated using Poisson regression analysis. Baseline characteristics of BP patients were compared across each year using analysis of variance, with 2019 data used as the reference group in post hoc comparisons via the Bonferroni method. All statistically significant levels were set at *p* < 0.05, and post‐hoc comparisons were considered significant when *p* < 0.01. All statistical analyses were performed using SAS version 9.4 (SAS Institute Inc., Cary, NC, USA) and R version 3.5.3 (R Foundation for Statistical Computing).

## 3 Results

### 3.1 Demographics

From 2017 to 2022, 103 482 patients were newly diagnosed with BP and included in the main analysis (Supporting Information S1: Table S3). Of these, 57 211 (55%) were men, and 46 271 (45%) were women. When the patients were divided according to the pre‐COVID (2017–2019), NPI (2020), vaccination (2021), and infection (2022) phases, 52 790 patients were diagnosed in the pre‐COVID, 17 591 during the NPI, 16 652 during the vaccination, and 16 449 during the infection phases.

Table 1 presents the demographic and baseline characteristics of the study population. The percentage of older patients ( ≥ 65 years) increased from 17.16% in 2017 to 20.42% in 2022. Compared to 2019, significant differences were observed in 2017 (*p* < 0.001) and 2022 (*p* = 0.009). The sex distribution remained relatively stable, with men consistently representing approximately 54%–56% of the cases. Notable trends in the prevalence of systemic diseases were observed. Hypertension, diabetes, dyslipidemia, hyperthyroidism, hypothyroidism, and autoimmune diseases showed an increasing trend over the years, whereas the prevalence of malignancy decreased over time. Overall, these data suggest an ageing population with an increasing burden of systemic comorbidities among those diagnosed with BP.

**Table 1:** Baseline characteristics of individuals diagnosed with Bell's palsy for each year.

	2017	2018	2019	2020	2021	2022	*p* a
	(*N* = 18 187)	(*N* = 17 564)	(*N* = 17 039)	(*N* = 17 591)	(*N* = 16 652)	(*N* = 16 449)	
Age (year)	*					*	< 0.001
0–19	689 (3.79)	447 (2.54)	451 (2.65)	538 (3.06)	445 (2.67)	387 (2.35)	
20–39	5176 (28.46)	4842 (27.57)	4558 (26.75)	4664 (26.51)	4288 (25.75)	4276 (26.00)	
40–64	9202 (50.60)	9005 (51.27)	8767 (51.45)	8912 (50.66)	8573 (51.48)	8427 (51.23)	
≥ 65	3120 (17.16)	3270 (18.62)	3263 (19.15)	3477 (19.77)	3346 (20.09)	3359 (20.42)	
Sex							0.005
Male	9856 (54.19)	9748 (55.50)	9334 (54.78)	9753 (55.44)	9311 (55.92)	9209 (55.99)	
Female	8331 (45.81)	7816 (44.50)	7705 (45.22)	7838 (44.56)	7341 (44.08)	7240 (44.01)	
Type of insurance							0.758
NHI	17687 (97.25)	17035 (96.99)	16551 (97.14)	17072 (97.05)	16179 (97.16)	15976 (97.12)	
Medical aid	500 (2.75)	529 (3.01)	488 (2.86)	519 (2.95)	473 (2.84)	473 (2.88)	
Systemic diseases							
Hypertension	4243 (23.33)*	4400 (25.05)*	4317 (25.34)	4455 (25.33)	4282 (25.71)	4319 (26.26)	< 0.001
Diabetes	2898 (15.93)*	3052 (17.38)*	2976 (17.47)	3227 (18.34)	3213 (19.29)*	3209 (19.51)*	< 0.001
Dyslipidemia	4101 (22.55)*	4627 (26.34)*	4935 (28.96)	5421 (30.82)*	5520 (33.15)*	5838 (35.49)*	< 0.001
Chronic kidney disease	141 (0.78)	133 (0.76)	157 (0.92)	162 (0.92)	168 (1.01)	166 (1.01)	0.038
Malignancy	1705 (9.37)	1623 (9.24)	1491 (8.75)	1430 (8.13)	1307 (7.85)*	1266 (7.70)*	< 0.001
Hyperthyroidism	225 (1.24)*	259 (1.47)	255 (1.50)	275 (1.56)	294 (1.77)*	306 (1.86)	< 0.001
Hypothyroidism	469 (2.58)*	561 (3.19)*	653 (3.83)	675 (3.84)	690 (4.14)*	744 (4.52)*	< 0.001
Chronic liver disease	892 (4.90)*	1088 (6.19)*	1192 (7.00)	1345 (7.65)*	1382 (8.30)*	1510 (9.18)*	< 0.001
Autoimmune diseases	1005 (5.53)*	1166 (6.64)*	1215 (7.13)	1478 (8.40)*	1517 (9.11)*	1566 (9.52)*	< 0.001

*Note:* Data are presented as *N* (%).

Abbreviations: CKD, chronic kidney disease; NHI, national health insurance.

^a^ *p* value from ANOVA; < 0.05 was considered statistically significant.

* *p* < 0.01 by post‐hoc Bonferroni test vs. 2019.

### 3.2 Trends of Monthly BP Incidence

**Figure 1:**
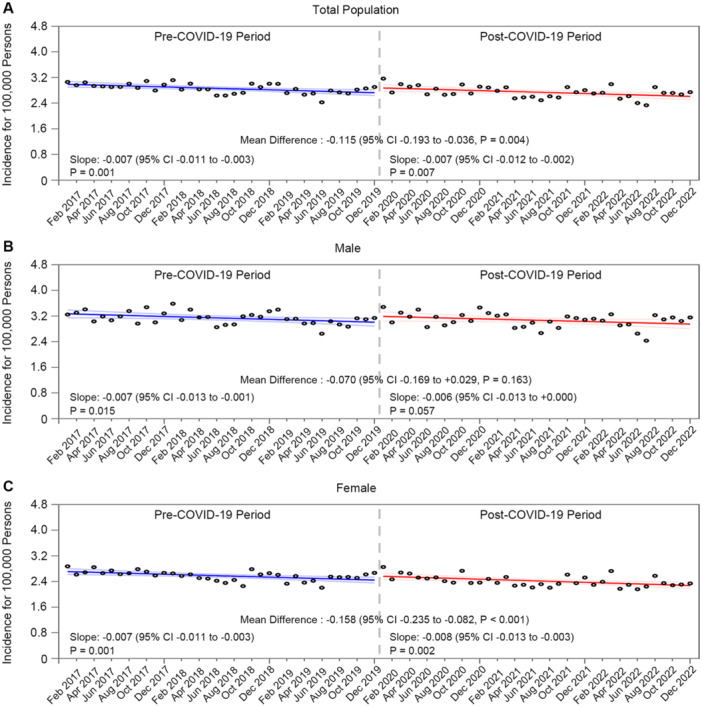
Trends in monthly Bell's Palsy incidence before and after the COVID‐19 outbreak by sex. The bold slope lines were estimated using the segmented regression mode, for (A) total population, (B) males, and (C) females. The bold blue line indicates the pre‐COVID‐19 period from 2017 to 2019, whereas the red bold line denotes the beginning of non‐pharmaceutical interventions for COVID‐19. The light blue and light red lines represent the 95% CI for the pre‐COVID‐19 and COVID‐19 periods, respectively. Abbreviations: BP, Bell's palsy; CI, confidence interval; COVID‐19, coronavirus disease‐19.

**Figure 2:**
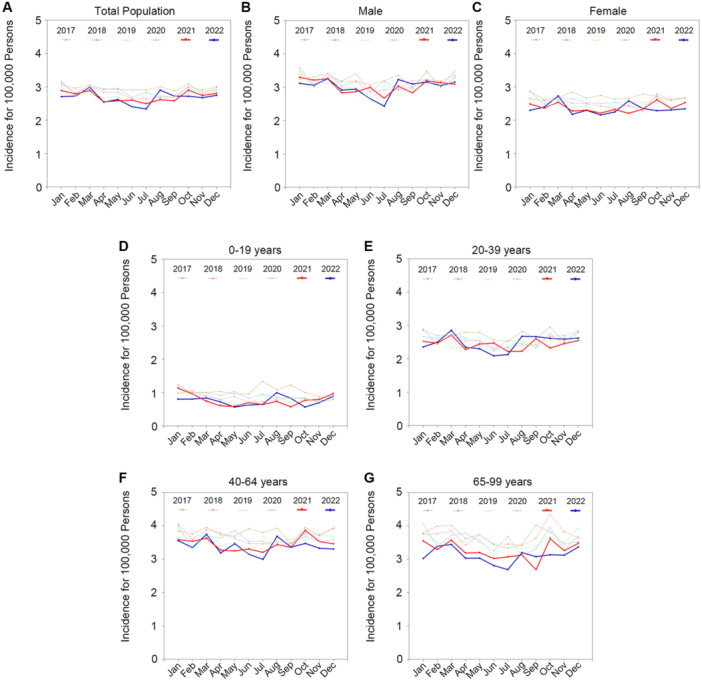
Monthly Bell's Palsy incidence from 2017 to 2022. The thick red line indicates the observed monthly BP incidence in 2021, while the thick blue line represents the observed incidence of BP in 2022 for the following groups: (A) total population, (B) males, (C) females, (D) individuals aged 0–19 years, (E) individuals aged 20–39 years, (F) individuals aged 40–64 years, and (G) individuals aged 65–99 years. Abbreviation: BP, Bell's palsy.

Estimates of trends of the monthly incidence of BP cases per 100 000 persons from January 2017 to December 2022 are presented in Figure 1 and Supporting Information S1: Figure S3. The monthly incidence of BP before the outbreak showed a decreasing trend (slope: −0.007, *p* for slope: 0.001). When the COVID‐19 pandemic began, the monthly incidence of BP declined further (slope: −0.007, *p* for slope: 0.007). The mean difference between pre‐ and post‐outbreak cases was −0.115 (95% CI: −0.193 to −0.036, *p* = 0.004). Following the pandemic, BP incidence significantly decreased among females (mean difference: −0.158, 95% CI: −0.235 to −0.082, *p*< 0.001) and across the 0–19‐ (mean difference: −0.087, 95% CI: −0.163 to −0.011, *p*= 0.025), 40–64‐ (mean difference: −0.210, 95% CI: −0.311 to −0.109, *p*< 0.001), and 65–99‐year (mean difference: −0.323, 95% CI: −0.459 to −0.188, *p*< 0.001) age groups. The monthly BP incidence from 2017 to 2022 by age groups and sex has been presented in Figure 2.

### 3.3 Comparison of BP Incidence Rates Among Phases

**Figure 3:**
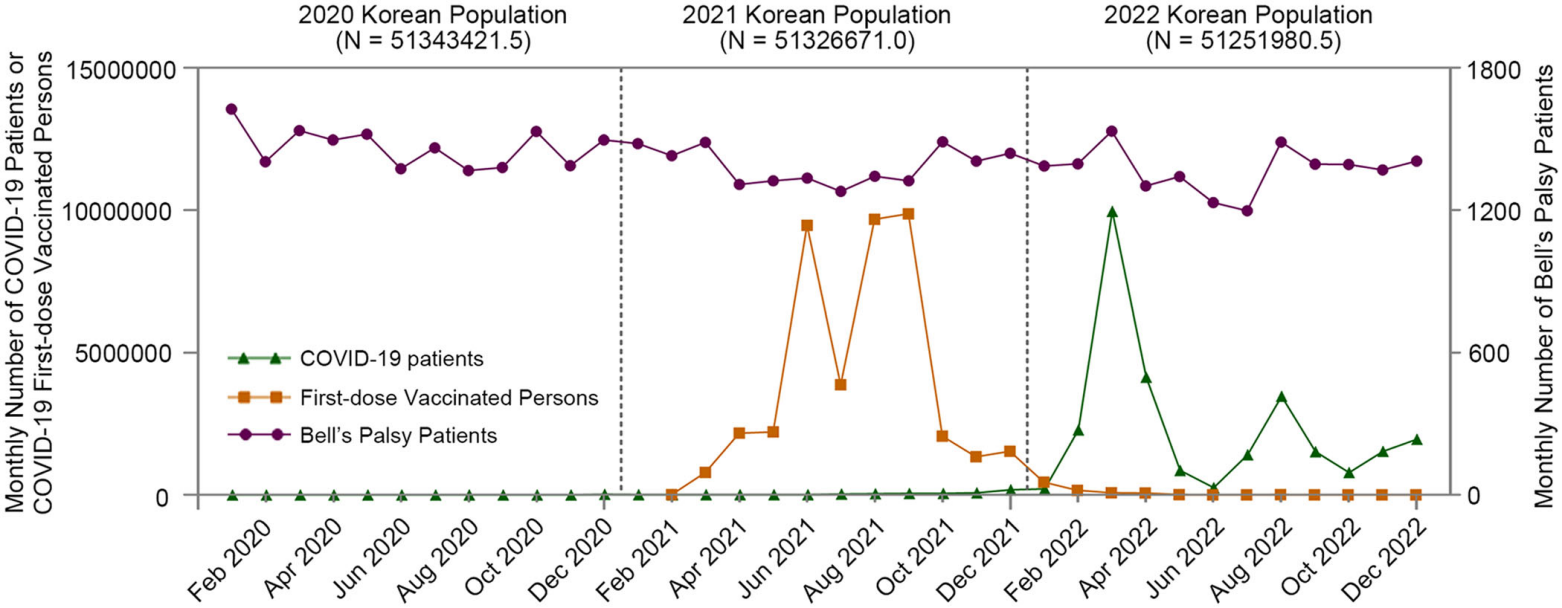
Changes in the monthly number of infections, vaccinations, and Bell's Palsy between 2020 and 2022 in Korea. The purple line with circle dots denotes the monthly number of patients with BP; the orange line with square dots denotes the number of vaccinated persons; and the green line with triangle dots denotes the number of SARS‐CoV‐2 infections. Abbreviations: BP, Bell's palsy; SARS‐CoV‐2, severe acute respiratory syndrome coronavirus 2.

The IRR of BP for the total study population showed a slight increase in 2020, with an age‐ and sex‐adjusted IRR of 1.02 (95% CI: 1.00–1.05, *p* = 0.026), demonstrating a significantly decreasing trend in 2021 and 2022 compared to 2019 (age‐ and sex‐adjusted IRR 0.96 [95% CI: 0.94–0.98, *p*< 0.001] for 2021; IRR 0.95 [95% CI: 0.93–0.97, *p*< 0.001] for 2022) (Table 2). The increase in BP incidence in 2020 appears to be primarily due to males (IRR 1.04 [95% CI: 1.01–1.07, *p*< 0.001]) and the age group below 19 years (IRR 1.13 [95% CI: 1.03–1.24, *p* = 0.008]) (Supporting Information S1: Table S4). The significant decrease in 2021 and 2022 appears to be primarily attributed to females (IRR 0.94 [95% CI: 0.91–0.97, *p*< 0.001] for 2021; IRR 0.92 [95% CI: 0.89–0.95, *p*< 0.001] for 2022) and the 40–64 (IRR 0.97 [95% CI: 0.94–0.99, *p* = 0.020] for 2021; IRR 0.95 [95% CI: 0.92–0.97, *p*< 0.001] for 2022) and 65–99 years age groups (IRR 0.92 [95% CI: 0.88–0.97, *p* = 0.001] for 2021; IRR 0.88 [95% CI: 0.84–0.93, *p*< 0.001] for 2022). The trends of BP incidence did not coincide with increases or decreases in vaccination or infection rates (Figure 3).

**Table 2:** Comparisons of the incidence rate ratio of Bell's palsy between pre‐ and post‐COVID‐19.

	Year	Residents	Events	Crude incidence rate (per 100 000 persons)	Unadjusted IRR (95% CI)	*p*	Age, sex‐adjusted IRR (95% CI)	*p*
Total	2017	51 226 047.0	18 187	35.50	1.07 (1.05–1.09)	< 0.001	1.09 (1.06–1.11)	< 0.001
	2018	51 295 997.0	17 564	34.24	1.03 (1.01–1.05)	0.004	1.04 (1.02–1.06)	< 0.001
	2019	51 332 197.0	17 039	33.19	1 (reference)		1 (reference)	
	2020	51 343 421.5	17 591	34.26	1.03 (1.01–1.05)	0.003	1.02 (1.00–1.05)	0.026
	2021	51 326 671.0	16 652	32.44	0.98 (0.96–1.00)	0.036	0.96 (0.94–0.98)	< 0.001
	2022	51 251 980.5	16 449	32.09	0.97 (0.95–0.99)	0.002	0.95 (0.93–0.97)	< 0.001
Males	2017	25 576 145.0	9856	38.54	1.06 (1.03–1.09)	< 0.001	1.07 (1.04–1.1.00)	< 0.001
	2018	25 601 323.0	9748	38.08	1.04 (1.02–1.07)	0.003	1.05 (1.02–1.08)	< 0.001
	2019	25 608 606.5	9334	36.45	1 (reference)		1 (reference)	
	2020	25 605 186.0	9753	38.09	1.05 (1.02–1.08)	0.002	1.04 (1.01–1.07)	0.011
	2021	25 588 082.0	9311	36.39	1.00 (0.97–1.03)	0.909	0.98 (0.96–1.01)	0.246
	2022	25 538 938.0	9209	36.06	0.99 (0.96–1.02)	0.464	0.97 (0.94–1.00)	0.028
Females	2017	25 649 902.0	8331	32.48	1.08 (1.05–1.12)	< 0.001	1.10 (1.07–1.14)	< 0.001
	2018	25 694 674.0	7816	30.42	1.02 (0.98–1.05)	0.337	1.02 (0.99–1.06)	0.141
	2019	25 723 590.5	7705	29.95	1 (reference)		1 (reference)	
	2020	25 738 235.5	7838	30.45	1.02 (0.99–1.05)	0.302	1.01 (0.98–1.04)	0.610
	2021	25 738 589.0	7341	28.52	0.95 (0.92–0.98)	0.003	0.94 (0.91–0.97)	< 0.001
	2022	25 713 042.5	7240	28.16	0.94 (0.91–0.97)	< 0.001	0.92 (0.89–0.95)	< 0.001

*Note:* IRR=annual mean incidence of Bell's palsy during the year/annual mean incidence during 2019.

Abbreviations: CI, confidence interval; IRR, incidence rate ratio.

*p* < 0.05 was considered statistically significant.

### 3.4 Sensitivity Analysis

In the sensitivity analysis, 150 861 patients were included: 78 287 patients in the pre‐COVID, 24 623 during the NPI, 24 192 during the vaccination, and 23 759 during the infection phases (Supporting Information S1: Table S5). The demographic and baseline characteristics are detailed in Supporting Information S1: Table S6. Estimates of trends of the monthly BP cases per 100 000 persons from 2017 to 2022 demonstrated similar results to those of the main study population (Supporting Information S1: Figures S4–S6). The mean difference between the pre‐ and post‐outbreak incidences was −0.311 (95% CI: −0.401 to −0.221, *p*< 0.001), which was significant in both men and women and in the 0–19, 40–64, and 65–99‐year age groups. The age‐ and sex‐adjusted IRR of BP for the total population showed a significant decreasing trend during the NPI, vaccination, and infection phases compared to the pre‐COVID‐19 phase (IRR 0.96 [95% CI: 0.94–0.97, *p*< 0.001] for 2020; IRR 0.93 [95% CI: 0.92–0.95, *p*< 0.001] for 2021; IRR 0.91 [95% CI: 0.89–0.93, *p*< 0.001] for 2022) (Supporting Information S1: Table S7). The significant decrease from 2020 to 2022 compared to 2019 was mainly attributed to females and the 40–64 and 65–99 years age groups (Supporting Information S1: Table S8), similar to the results of the main study population. The trends in BP incidence did not correlate with vaccination or infection rates (Supporting Information S1: Figure S7).

## 4 Discussion

In this ecological study, we investigated the association between population‐level exposure to NPI (2020), COVID‐19 vaccination (2021), and infection (2022), and the incidence of Bell's palsy (BP) using data from the HIRA database. The results indicated that the nationwide incidence of BP in 2021 and 2022 was lower than the pre‐outbreak rates and there was no evident increase in BP incidence during either the vaccination or infection phases. However, during this period, an increasing trend in the prevalence of known risk factors for Bell's palsy—such as hypertension, diabetes, and hyperlipidemia—was observed among patients diagnosed with BP. This may be partly attributable to government‐led social distancing measures, as well as possible changes in dietary and physical activity habits [10].

Several studies have investigated the potential association between COVID‐19 vaccines and Bell's palsy (BP). While some research has suggested links between BP and specific vaccine types—such as mRNA or inactivated virus vaccines—findings have been inconsistent. For example, a 2022 study reported an increased BP risk after CoronaVac vaccination but no association with mRNA vaccines, though the low vaccination rate in the control group may have limited its reliability [11, 12]. Another study using WHO pharmacovigilance data suggested a possible association between mRNA vaccines and facial paralysis (reported odds ratio, 28.31); however, similar associations were also observed for most other vaccines, except for the tuberculosis vaccine [13]. A South Korean self‐controlled case series in 2024 also showed a slight increase in BP risk postvaccination (IRR, 1.12) [14], but subsequent large‐scale studies, including our own involving over 8 million individuals, found no such increase [15]. Meta‐analyses and systematic reviews have further supported these findings, suggesting that the incidence of BP following COVID‐19 vaccination is likely comparable to that in unvaccinated populations [16, 17]. Collectively, these results suggest that a causal relationship between COVID‐19 vaccination and BP has not been clearly established.

The central nervous system (CNS) can be infected by SARS‐CoV‐2 through hematogenous spread, as angiotensin‐converting enzyme 2 (ACE2) receptors—utilized by the virus for cellular entry—are also expressed in the CNS [18, 19]. Furthermore, hyperinflammatory responses following SARS‐CoV‐2 infection have been associated with various complications, including thromboembolism and stroke, underscoring the broader systemic effects of immune dysregulation [20, 21]. These mechanisms suggest that both direct viral neurotropism and indirect immune‐mediated pathways may contribute to the development of Bell's palsy (BP) in some individuals.

Based on these theoretical grounds, several studies have explored the potential association between SARS‐CoV‐2 infection and BP. A large South Korean cohort study involving over 48 million individuals reported a significant association, with an adjusted subdistribution hazard ratio of 1.24 [6]. Similarly, a U.S.‐based retrospective longitudinal study identified an increased BP risk among patients with COVID‐19 [7]. In contrast, other studies have yielded differing results. For example, a retrospective cohort study including 22,658 patients found no association between SARS‐CoV‐2 infection and BP risk [22], and a single‐center study conducted at a tertiary care hospital also reported no clear link between COVID‐19 and BP [23].

Existing studies on this topic have presented sharply conflicting viewpoints, so the findings of the present study may contribute additional insights into this debate. During the COVID‐19 pandemic, BP incidence rates declined during the vaccination and infection phases compared with the control period in the general population. Several factors may explain this trend. First, improvement in public hygiene and the widespread adoption of NPIs such as social distancing and mask‐wearing decreased the transmission of viral infections that could trigger BP. Viruses such as HSV, varicella‐zoster virus (VZV), and Epstein‐Barr virus (EBV) inflame or damage the facial nerve, impairing its peripheral function and leading to temporary paralysis. Second, relative immunosuppression is a potential factor in BP etiology, as it can reactivate latent virus in the geniculate ganglia [24]. However, SARS‐CoV‐2 vaccination and infection lead to both humoral and cell‐mediated immunity activation, which may help prevent such viral reactivation. Third, restricted access to healthcare during the pandemic led to individuals' avoidance of medical visits for nonemergency conditions, resulting in fewer BP diagnoses, even with stable incidence rates. Various factors seem to be involved in the pathophysiology of the association, and further investigations are required.

According to the demographic data, the number of patients diagnosed with BP decreased in 2021 and 2022; however, the proportion of older adult patients aged ≥ 65 years increased. Consequently, the likelihood of developing systemic diseases also appears to have increased, with particularly high rates of hypertension, diabetes, and dyslipidemia, which worsen BP prognosis. Patients with concomitant comorbidities generally have poorer outcomes compared with those without comorbidities. Increased glycosylated hemoglobin A1c levels ( > 6.7%) are significantly correlated with unsatisfactory facial recovery and a higher likelihood of severe facial nerve degeneration. Diabetes, hypercholesterolemia, and hypertension also affect the vasa nervorum of the facial nerve [10, 25]. Additionally, the present analysis revealed a statistically significant decrease in IRR in 2021 and 2022, particularly among women and individuals aged over 40 years. However, this does not suggest a significant increase in IRR among men or other age groups. Given these complex and multifactorial influences, BP risk during the pandemic should be interpreted cautiously. Patient counselling should reflect individual risk profiles rather than promote generalized concerns regarding vaccination or SARS‐CoV‐2 infection. Continued research is warranted to clarify the interplay between viral infections, immune responses, and BP development in both pandemic and non‐pandemic contexts.

Our study had several limitations. First, it relied on diagnostic and procedural codes using healthcare claims and KCD codes for analysis, which can lead to misclassification or underdiagnosis, although conditions that could mimic BP were excluded. Additionally, the primary BP definition, which required corticosteroid prescriptions, might have led to an underestimation of cases, as some cases may not have received medication. However, this did not affect the overall trends, as sensitivity analysis with an alternative definition supported the study's main findings. Second, although multiple SARS‐CoV‐2 variants and vaccines emerged during the study period, specific types of variants or vaccines could not be differentiated because of the nature of the study. This study could be enhanced by future research comparing BP incidence based on the different types of viral variants and vaccines. Third, findings such as neurologic symptoms, imaging studies, and laboratory testing could not be assessed. Lastly, there is a potential for ecological fallacy, in which associations observed at the population level may not accurately reflect those at the individual level. As this study was based on aggregated national data, we were unable to determine whether individuals who developed BP were directly exposed to SARS‐CoV‐2 infection or vaccination. Although we analyzed temporal trends across distinct pandemic phases, the lack of individual‐level exposure data limited our ability to establish clear causal relationships between exposure and outcome. Nevertheless, considering that 87% of the total population in South Korea received at least two doses of a COVID‐19 vaccine in 2021, and that 60% were infected in 2022, this ecological study suggests that the impact of COVID‐19 vaccination or infection on the incidence of Bell's palsy (BP) is likely to be limited.

In conclusion, despite these limitations, this study provides meaningful insights by leveraging the unique characteristics of the South Korean population. It is the largest study of its kind in South Korea, with long‐term analyses covering the pre‐COVID‐19, NPI, vaccination, and infection phases, enabling a comprehensive examination of BP incidence. The findings may provide helpful context for future research and contribute to ongoing discussions about BP risk in the COVID‐19 context.

## Author Contributions

Seungyeon Lee and Yong Joon Kim contributed to the study design, statistical analysis plan, data analysis, and interpretation of results. Seungyeon Lee contributed to manuscript writing and the development of the outline. Seung Won Lee and Yong Joon Kim participated in the review of the study analysis plan and critically reviewed the results. Nang Kyeong Lee and Seung Won Lee planned the statistical analysis, data management, and ran the analysis. All co‐authors reviewed the manuscript and agreed with its content.

## Ethics Statement

This nationwide, population‐based ecological study was approved by the Institutional Review Board of Yonsei University Severance Hospital (approval number 4‐2024‐1293), and the Korean Health Insurance Review and Assessment (HIRA) Deliberative Committee approved the conditional use of the de‐identified data (approval number M20230828002).

### 1 Consent

The study adhered to the tenets of the Declaration of Helsinki, and the requirement for written informed consent was waived because all data provided by the HIRA were anonymized.

## Conflicts of Interest

The authors declare no conflicts of interest.

## Supporting information

Supporting Materials R1.

## Data Availability

The data sets generated and/or analyzed during the current study are not publicly available due to data use restrictions under the policies of the Health Insurance Review and Assessment Service (HIRA). However, qualified researchers can apply to HIRA to construct the same data set by submitting a research proposal and obtaining approval from an Institutional Review Board (IRB). Upon receiving HIRA's permission and paying the required data access fees, researchers can proceed with their studies. For more information, please refer to HIRA's Open Data Portal: https://opendata.hira.or.kr.
