# Supplementary material for: Changes in the Nationwide Incidence of Bell's Palsy in the General Population Before and After the COVID‐19 Pandemic: An Ecological Study
Source: J Med Virol. 2025 Jun 2;97(6):e70431. doi: 10.1002/jmv.70431 (PMC12128729; doi:10.1002/jmv.70431)
Supplement: Supplementary file 1 — Supporting Materials R1. [file JMV-97-e70431-s001.docx]

**ONLINE SUPPLEMENTAL MATERIALS**

**Changes in the Nationwide Incidence of Bell’s Palsy in the General Population Before and After the COVID-19 Pandemic: An Ecological Study**

Seungyeon Lee, MD^1^, Nang Kyeong Lee, MSc^2^, Seung Won Lee, MD, PhD^2*^, Yong Joon Kim, MD, PhD^1*^

^1^ Department of Ophthalmology, Institute of Vision Research, Yonsei University College of Medicine, Seoul, Republic of Korea

^2^ Department of Precision Medicine, School of Medicine, Sungkyunkwan University, Suwon, Republic of Korea

^*^ Y.J. Kim and S.W. Lee equally contributed to this work as co-corresponding authors.

**Supplementary Table S1.** Definitions of drug treatments and KCD-8 codes to identify Bell’s palsy and other comorbidities.

|  | **Definition** | **Condition** |
| --- | --- | --- |
| **Medical conditions relevant to the diagnosis of Bell’s palsy** | | |
| Bell’s palsy | Defined from diagnosis* | KCD-8: G510 |
| Clonic hemifacial spasm | Defined from diagnosis* | KCD-8: G513 |
| Facial myokymia | Defined from diagnosis* | KCD-8: G514 |
| Other disorders of facial nerve | Defined from diagnosis* | KCD-8: G511, G512, G518, G519 |
| Acute myocardial infarction | Defined from diagnosis* | KCD-8: I21, I22, I25.2 |
| Hemorrhagic stroke | Defined from diagnosis* | KCD-8: I60, I61, I62 |
| Ischemic stroke | Defined from diagnosis* | KCD-8: I63, I64 |
| Injury of facial nerve | Defined from diagnosis* | KCD-8: S045 |
| Intracranial injury | Defined from diagnosis* | KCD-8: S06, T905 |
| Herpes zoster | Defined from diagnosis* | KCD-8: B02 |
| **Steroid treatment for Bell’s palsy treatment** | | |
| Injection | Prescription within 60 days of Bell’s palsy diagnosis | ATC code: 116502BIJ, 142001BIJ, 142201BIJ, 142202BIJ, 142203BIJ, 171201BIJ, 171202BIJ, 171203BIJ, 193501BIJ, 193502BIJ, 193601BIJ, 193602BIJ, 193603BIJ, 193604BIJ, 193605BIJ, 217101BIJ, 217104BIJ, 217301BIJ, 217302BIJ, 243301BIJ, 243303BIJ, 243305BIJ, 316100BIJ, 316200BIJ |
| Suppository | Prescription within 60 days of Bell’s palsy diagnosis | ATC code: 337200CSP, 338000CSP, 338300CSP, 338400CSP |
| Tablet | Prescription within 60 days of Bell’s palsy diagnosis | ATC code: 116401ATB, 116501ATB, 141901ATB, 141903ATB, 141904ATB, 170901ATB, 170905ATB, 170906ATB, 171204ATB, 193301ATB, 193302ATB, 193303ATB, 193304ATB, 193305ATB, 217001ATB, 217401ATB, 243201ATB, 243202ATB, 243203ATB, 296900ATB |
| Liquid | Prescription within 60 days of Bell’s palsy diagnosis | ATC code: 116601CLQ, 170901CLQ, 170902CLQ, 170903CLQ, 217002CLQ, 243309CLQ, 243309CLQ, 334100CLQ, 334200CLQ, 347600CLQ |
| Syrup | Prescription within 60 days of Bell’s palsy diagnosis | ATC code: 217003ASY, 217004ASY |

| **Comorbidities** |  |  |
| --- | --- | --- |
| Hypertension | Defined from diagnosis* | KCD-8: I10, I11, I12, I13, I15 |
| Diabetes mellitus | Defined from diagnosis* | KCD-8: E10, E11, E12, E13, E14 |
| Dyslipidemia | Defined from diagnosis* | KCD-8: E78 |
| Chronic kidney disease | Defined from diagnosis* | KCD-8: N18, N19 |
| Malignancy | Defined from diagnosis* | KCD-8: C00-C97 |
| Hyperthyroidism | Defined from diagnosis* | KCD-8: E05 |
| Hypothyroidism | Defined from diagnosis* | KCD-8: E03 |
| Chronic liver disease | Defined from diagnosis* | KCD-8: B18, K70, K71, K72, K73, K74, K76.1 |
| Autoimmune diseases | Defined from diagnosis* | KCD-8: D686, D86, M023, M028, M029, M05, M06, M07, M08, M09, M30, M31, M32, M33, M34, M35, M364, M45, M469, M941, K754, E063, K51, K50, L40 |

*Comorbidities were established based on one inpatient or two outpatient records of KCD-8 codes in the database. Abbreviations: ATC, Anatomical Therapeutic Chemical Classification System; KCD-8, Korean Standard Classification of Diseases, 8th Revision

**Supplementary Table S2.** Mid-year population of Korea for the study period.

| **Total** | | | | | | |
| --- | --- | --- | --- | --- | --- | --- |
| **Age (y)** | **2017** | **2018** | **2019** | **2020** | **2021** | **2022** |
| 0–4 | 2141131.5 | 2026168.5 | 1909182.5 | 1760637.5 | 1603407.5 | 1478729.0 |
| 5–9 | 2356869.5 | 2340328.0 | 2323178.5 | 2305388.5 | 2259652.5 | 2165857.0 |
| 10–14 | 2346220.0 | 2334187.0 | 2308928.5 | 2314984.0 | 2347218.0 | 2357797.0 |
| 15–19 | 3034107.5 | 2872026.0 | 2725294.5 | 2551012.5 | 2395066.0 | 2340932.5 |
| 20–24 | 3498959.0 | 3436410.5 | 3353801.0 | 3272265.0 | 3175015.0 | 3023612.0 |
| 25–29 | 3252272.5 | 3347964.5 | 3430877.0 | 3505179.5 | 3529975.0 | 3493108.5 |
| 30–34 | 3389545.5 | 3227142.5 | 3151167.0 | 3130579.0 | 3162503.5 | 3252002.5 |
| 35–39 | 3999098.5 | 4031400.0 | 3960321.0 | 3786437.5 | 3590685.5 | 3383872.0 |
| 40–44 | 4104695.0 | 3959875.0 | 3857129.0 | 3863754.5 | 3924880.0 | 3982294.0 |
| 45–49 | 4535798.0 | 4531587.5 | 4478729.5 | 4380758.0 | 4227626.5 | 4078442.5 |
| 50–54 | 4105674.0 | 4155086.0 | 4258855.0 | 4331294.0 | 4422823.0 | 4495141.0 |
| 55–59 | 4224127.5 | 4273105.0 | 4259891.5 | 4207585.0 | 4118758.5 | 4053405.5 |
| 60–64 | 3176004.5 | 3376125.5 | 3601453.0 | 3804709.5 | 3997811.5 | 4143412.0 |
| 65–69 | 2262097.0 | 2345864.5 | 2443839.5 | 2635592.0 | 2881870.0 | 3087466.0 |
| 70–74 | 1765782.0 | 1814165.0 | 1902232.0 | 2000708.5 | 2075136.5 | 2148314.0 |
| 75–79 | 1506433.5 | 1585356.0 | 1603245.5 | 1602662.5 | 1597970.0 | 1604258.5 |
| 80–84 | 928484.5 | 989634.5 | 1056165.0 | 1110911.5 | 1163257.5 | 1247806.0 |
| 85–89 | 426388.0 | 460471.5 | 499619.0 | 549650.5 | 603274.5 | 647492.0 |
| 90–94 | 140858.0 | 153059.5 | 169218.5 | 188043.5 | 205711.0 | 221408.0 |
| 95–99 | 31501.0 | 36040.0 | 39069.5 | 41268.5 | 44029.0 | 46630.5 |
| Sum (0–99) | 51226047.0 | 51295997.0 | 51332197.0 | 51343421.5 | 51326671.0 | 51251980.5 |
| **Males** | | | | | | |
| **Age (y)** | **2017** | **2018** | **2019** | **2020** | **2021** | **2022** |
| 0–4 | 1097772.5 | 1039018.0 | 979211.0 | 903178.0 | 822670.5 | 758337.0 |
| 5–9 | 1212386.0 | 1203026.0 | 1193014.0 | 1182550.5 | 1158010.5 | 1109783.0 |
| 10–14 | 1215944.0 | 1206456.0 | 1191290.5 | 1192976.5 | 1208122.5 | 1212408.0 |
| 15–19 | 1582678.5 | 1497710.0 | 1418938.0 | 1325906.0 | 1242509.0 | 1211372.5 |
| 20–24 | 1851296.0 | 1810584.5 | 1759184.0 | 1709726.0 | 1654306.5 | 1574159.0 |
| 25–29 | 1705353.0 | 1762115.0 | 1811314.5 | 1852731.0 | 1864933.5 | 1841935.5 |
| 30–34 | 1740691.5 | 1661315.0 | 1627594.0 | 1623891.5 | 1646546.5 | 1698629.5 |
| 35–39 | 2037572.5 | 2054181.0 | 2018361.5 | 1933535.5 | 1836896.0 | 1733721.0 |
| 40–44 | 2082042.0 | 2009430.0 | 1959709.0 | 1962337.0 | 1995164.0 | 2026228.0 |
| 45–49 | 2294774.5 | 2299107.0 | 2274031.5 | 2224690.0 | 2145192.0 | 2065095.5 |
| 50–54 | 2088050.5 | 2100952.5 | 2146978.0 | 2184220.0 | 2228227.0 | 2266516.5 |
| 55–59 | 2110123.0 | 2138816.5 | 2136895.0 | 2115455.0 | 2081329.5 | 2049472.0 |
| 60–64 | 1561178.5 | 1665402.5 | 1778062.5 | 1878929.5 | 1976075.0 | 2050119.0 |
| 65–69 | 1087298.0 | 1128819.0 | 1177940.0 | 1271779.5 | 1390963.5 | 1494388.5 |
| 70–74 | 803598.5 | 834873.5 | 883751.0 | 934279.5 | 970430.0 | 1006369.0 |
| 75–79 | 623318.0 | 662915.5 | 675845.0 | 682114.5 | 688442.0 | 700270.5 |
| 80–84 | 328109.0 | 356227.0 | 386770.5 | 413379.5 | 439372.0 | 478563.0 |
| 85–89 | 116596.5 | 129511.5 | 144408.5 | 163454.0 | 184245.5 | 202017.5 |
| 90–94 | 31290.5 | 33835.0 | 37636.0 | 42101.5 | 46457.5 | 51075.5 |
| 95–99 | 6072.0 | 7027.5 | 7672.0 | 7951.0 | 8189.0 | 8477.5 |
| Sum (0–99) | 25576145.0 | 25601323.0 | 25608606.5 | 25605186.0 | 25588082.0 | 25538938.0 |
| **Females** | | | | | | |
| **Age (y)** | **2017** | **2018** | **2019** | **2020** | **2021** | **2022** |
| 0–4 | 1043359.0 | 987150.5 | 929971.5 | 857459.5 | 780737.0 | 720392.0 |
| 5–9 | 1144483.5 | 1137302.0 | 1130164.5 | 1122838.0 | 1101642.0 | 1056074.0 |
| 10–14 | 1130276.0 | 1127731.0 | 1117638.0 | 1122007.5 | 1139095.5 | 1145389.0 |
| 15–19 | 1451429.0 | 1374316.0 | 1306356.5 | 1225106.5 | 1152557.0 | 1129560.0 |
| 20–24 | 1647663.0 | 1625826.0 | 1594617.0 | 1562539.0 | 1520708.5 | 1449453.0 |
| 25–29 | 1546919.5 | 1585849.5 | 1619562.5 | 1652448.5 | 1665041.5 | 1651173.0 |
| 30–34 | 1648854.0 | 1565827.5 | 1523573.0 | 1506687.5 | 1515957.0 | 1553373.0 |
| 35–39 | 1961526.0 | 1977219.0 | 1941959.5 | 1852902.0 | 1753789.5 | 1650151.0 |
| 40–44 | 2022653.0 | 1950445.0 | 1897420.0 | 1901417.5 | 1929716.0 | 1956066.0 |
| 45–49 | 2241023.5 | 2232480.5 | 2204698.0 | 2156068.0 | 2082434.5 | 2013347.0 |
| 50–54 | 2017623.5 | 2054133.5 | 2111877.0 | 2147074.0 | 2194596.0 | 2228624.5 |
| 55–59 | 2114004.5 | 2134288.5 | 2122996.5 | 2092130.0 | 2037429.0 | 2003933.5 |
| 60–64 | 1614826.0 | 1710723.0 | 1823390.5 | 1925780.0 | 2021736.5 | 2093293.0 |
| 65–69 | 1174799.0 | 1217045.5 | 1265899.5 | 1363812.5 | 1490906.5 | 1593077.5 |
| 70–74 | 962183.5 | 979291.5 | 1018481.0 | 1066429.0 | 1104706.5 | 1141945.0 |
| 75–79 | 883115.5 | 922440.5 | 927400.5 | 920548.0 | 909528.0 | 903988.0 |
| 80–84 | 600375.5 | 633407.5 | 669394.5 | 697532.0 | 723885.5 | 769243.0 |
| 85–89 | 309791.5 | 330960.0 | 355210.5 | 386196.5 | 419029.0 | 445474.5 |
| 90–94 | 109567.5 | 119224.5 | 131582.5 | 145942.0 | 159253.5 | 170332.5 |
| 95–99 | 25429.0 | 29012.5 | 31397.5 | 33317.5 | 35840.0 | 38153.0 |
| Sum (0–99) | 25649902.0 | 25694674.0 | 25723590.5 | 25738235.5 | 25738589.0 | 25713042.5 |

**Supplementary Table S3.** Annual incidence of Bell’s palsy between 2017 and 2022 in South Korea.

| Age (years) | 2017 | | | 2018 | | | 2019 | | | 2020 | | | 2021 | | | 2022 | | |
| --- | --- | --- | --- | --- | --- | --- | --- | --- | --- | --- | --- | --- | --- | --- | --- | --- | --- | --- |
|  | M | F | Total | M | F | Total | M | F | Total | M | F | Total | M | F | Total | M | F | Total |
| 0–4 | 99 | 107 | 206 | 39 | 43 | 82 | 33 | 42 | 75 | 22 | 22 | 44 | 24 | 29 | 53 | 27 | 31 | 58 |
| 5–9 | 85 | 82 | 167 | 56 | 56 | 112 | 52 | 57 | 109 | 51 | 60 | 111 | 48 | 48 | 96 | 34 | 43 | 77 |
| 10–14 | 181 | 135 | 316 | 133 | 120 | 253 | 132 | 135 | 267 | 203 | 180 | 383 | 159 | 137 | 296 | 131 | 121 | 252 |
| 15–19 | 299 | 253 | 552 | 271 | 240 | 511 | 216 | 231 | 447 | 236 | 205 | 441 | 185 | 160 | 345 | 194 | 167 | 361 |
| 20–24 | 399 | 350 | 749 | 400 | 364 | 764 | 373 | 299 | 672 | 352 | 352 | 704 | 332 | 334 | 666 | 355 | 289 | 644 |
| 25–29 | 578 | 411 | 989 | 587 | 390 | 977 | 580 | 380 | 960 | 609 | 419 | 1,028 | 562 | 381 | 943 | 530 | 425 | 955 |
| 30–34 | 745 | 521 | 1,266 | 640 | 447 | 1,087 | 577 | 445 | 1,022 | 665 | 475 | 1,140 | 614 | 405 | 1,019 | 661 | 412 | 1,073 |
| 35–39 | 958 | 662 | 1,620 | 939 | 564 | 1,503 | 913 | 544 | 1,457 | 837 | 514 | 1,351 | 795 | 520 | 1,315 | 757 | 486 | 1,243 |
| 40–44 | 1,059 | 647 | 1,706 | 973 | 567 | 1,540 | 928 | 542 | 1,470 | 959 | 572 | 1,531 | 922 | 599 | 1,521 | 925 | 556 | 1,481 |
| 45–49 | 1,140 | 740 | 1,880 | 1,146 | 727 | 1,873 | 1,075 | 728 | 1,803 | 1,125 | 672 | 1,797 | 1,016 | 629 | 1,645 | 963 | 636 | 1,599 |
| 50–54 | 1,049 | 881 | 1,930 | 1,074 | 864 | 1,938 | 1,007 | 838 | 1,845 | 1,049 | 818 | 1,867 | 1,072 | 771 | 1,843 | 1,061 | 804 | 1,865 |
| 55–59 | 1,149 | 934 | 2,083 | 1,083 | 879 | 1,962 | 1,099 | 875 | 1,974 | 1,147 | 860 | 2,007 | 1,042 | 769 | 1,811 | 977 | 752 | 1,729 |
| 60–64 | 804 | 799 | 1,603 | 907 | 785 | 1,692 | 893 | 782 | 1,675 | 929 | 781 | 1,710 | 968 | 785 | 1,753 | 984 | 769 | 1,753 |
| 65–69 | 486 | 611 | 1,097 | 596 | 557 | 1,153 | 578 | 598 | 1,176 | 623 | 635 | 1,258 | 687 | 650 | 1,337 | 696 | 626 | 1,322 |
| 70–74 | 358 | 510 | 868 | 405 | 453 | 858 | 403 | 436 | 839 | 436 | 510 | 946 | 427 | 452 | 879 | 429 | 423 | 852 |
| 75–79 | 310 | 368 | 678 | 302 | 412 | 714 | 281 | 425 | 706 | 301 | 395 | 696 | 252 | 334 | 586 | 263 | 334 | 597 |
| 80–84 | 123 | 215 | 338 | 144 | 228 | 372 | 138 | 221 | 359 | 157 | 241 | 398 | 141 | 228 | 369 | 137 | 233 | 370 |
| 85–89 | 24 | 82 | 106 | 48 | 93 | 141 | 49 | 100 | 149 | 46 | 92 | 138 | 51 | 89 | 140 | 69 | 90 | 159 |
| 90–94 | 8 | 19 | 27 | 4 | 22 | 26 | 6 | 21 | 27 | 5 | 31 | 36 | 13 | 17 | 30 | 14 | 37 | 51 |
| 95–99 | 2 | 4 | 6 | 1 | 5 | 6 | 1 | 6 | 7 | 1 | 4 | 5 | 1 | 4 | 5 | 2 | 6 | 8 |
| Total | 9,856 | 8,331 | 18,187 | 9,748 | 7,816 | 17,564 | 9,334 | 7,705 | 17,039 | 9,753 | 7,838 | 17,591 | 9,311 | 7,341 | 16,652 | 9,209 | 7,240 | 16,449 |

Abbreviations: M, males; F, females.

**Supplementary Table S4.** Comparisons of incidence rate ratio of Bell’s palsy between pre- and post-COVID-19. Age group

| Age group | Year | Residents | Events | Crude incidence rate (per 100,000 persons) |  | Unadjusted IRR  (95% CI) | P |  | Age, sex-adjusted IRR (95% CI) | P |
| --- | --- | --- | --- | --- | --- | --- | --- | --- | --- | --- |
| 0–19 | 2017 | 9878328.5 | 1,241 | 12.56 |  | 1.30 (1.19–1.41) | <0.001 |  | 1.29 (1.18–1.4) | <0.001 |
|  | 2018 | 9572709.5 | 958 | 10.01 |  | 1.03 (0.94–1.13) | 0.488 |  | 1.03 (0.94–1.13) | 0.537 |
|  | 2019 | 9266584.0 | 898 | 9.69 |  | 1 (reference) |  |  | 1 (reference) |  |
|  | 2020 | 8932022.5 | 979 | 10.96 |  | 1.13 (1.03–1.24) | 0.008 |  | 1.13 (1.03–1.24) | 0.008 |
|  | 2021 | 8605344.0 | 790 | 9.18 |  | 0.95 (0.86–1.04) | 0.267 |  | 0.94 (0.86–1.04) | 0.246 |
|  | 2022 | 8343315.5 | 748 | 8.97 |  | 0.93 (0.84–1.02) | 0.116 |  | 0.92 (0.83–1.01) | 0.080 |
| 20–39 | 2017 | 14139875.5 | 4,624 | 32.70 |  | 1.11 (1.06–1.15) | <0.001 |  | 1.11 (1.06–1.15) | <0.001 |
|  | 2018 | 14042917.5 | 4,331 | 30.84 |  | 1.04 (1.00–1.09) | 0.056 |  | 1.04 (1.00–1.09) | 0.056 |
|  | 2019 | 13896166.0 | 4,111 | 29.58 |  | 1 (reference) |  |  | 1 (reference) |  |
|  | 2020 | 13694461.0 | 4,223 | 30.84 |  | 1.04 (1.00–1.09) | 0.058 |  | 1.04 (1.00–1.09) | 0.053 |
|  | 2021 | 13458179.0 | 3,943 | 29.30 |  | 0.99 (0.95–1.03) | 0.663 |  | 0.99 (0.95–1.04) | 0.757 |
|  | 2022 | 13152595.0 | 3,915 | 29.77 |  | 1.01 (0.96–1.05) | 0.783 |  | 1.01 (0.96–1.05) | 0.721 |
| 40–64 | 2017 | 20146299.0 | 9,202 | 45.68 |  | 1.07 (1.04–1.10) | <0.001 |  | 1.07 (1.04–1.10) | <0.001 |
|  | 2018 | 20295779.0 | 9,005 | 44.37 |  | 1.04 (1.01–1.07) | 0.021 |  | 1.04 (1.01–1.07) | 0.014 |
|  | 2019 | 20456058.0 | 8,767 | 42.86 |  | 1 (reference) |  |  | 1 (reference) |  |
|  | 2020 | 20588101.0 | 8,912 | 43.29 |  | 1.01 (0.98–1.04) | 0.507 |  | 1.01 (0.98–1.04) | 0.538 |
|  | 2021 | 20691899.5 | 8,573 | 41.43 |  | 0.97 (0.94–1.00) | 0.026 |  | 0.97 (0.94–0.99) | 0.020 |
|  | 2022 | 20752695.0 | 8,427 | 40.61 |  | 0.95 (0.92–0.98) | <0.001 |  | 0.95 (0.92–0.97) | <0.001 |
| 65–99 | 2017 | 7061544.0 | 3,120 | 44.18 |  | 1.04 (0.99–1.10) | 0.082 |  | 1.04 (0.99–1.09) | 0.125 |
|  | 2018 | 7384591.0 | 3,270 | 44.28 |  | 1.05 (1.00–1.10) | 0.065 |  | 1.04 (1.00–1.10) | 0.078 |
|  | 2019 | 7713389.0 | 3,263 | 42.30 |  | 1 (reference) |  |  | 1 (reference) |  |
|  | 2020 | 8128837.0 | 3,477 | 42.77 |  | 1.01 (0.96–1.06) | 0.650 |  | 1.01 (0.96–1.06) | 0.621 |
|  | 2021 | 8571248.5 | 3,346 | 39.04 |  | 0.92 (0.88–0.97) | 0.001 |  | 0.92 (0.88–0.97) | 0.001 |
|  | 2022 | 9003375.0 | 3,359 | 37.31 |  | 0.88 (0.84–0.93) | <0.001 |  | 0.88 (0.84–0.93) | <0.001 |

^*^IRR=annual mean incidence of Bells’ palsy during the year/annual mean incidence in 2019.

P<0.05 was considered statistically significant.

Abbreviations: IRR, incidence rate ratio; CI, confidence interval.

**Supplementary Table S5.** Sensitivity analysis of the annual incidence of Bell’s palsy between 2017 and 2022 in South Korea.

| Age (years) | 2017 | | | 2018 | | | 2019 | | | 2020 | | | 2021 | | | 2022 | | |
| --- | --- | --- | --- | --- | --- | --- | --- | --- | --- | --- | --- | --- | --- | --- | --- | --- | --- | --- |
|  | M | F | Total | M | F | Total | M | F | Total | M | F | Total | M | F | Total | M | F | Total |
| 0–4 | 176 | 185 | 361 | 98 | 99 | 197 | 111 | 108 | 219 | 84 | 75 | 159 | 83 | 79 | 162 | 86 | 81 | 167 |
| 5–9 | 112 | 106 | 218 | 83 | 84 | 167 | 77 | 91 | 168 | 74 | 86 | 160 | 66 | 76 | 142 | 53 | 58 | 111 |
| 10–14 | 221 | 167 | 388 | 173 | 150 | 323 | 174 | 158 | 332 | 237 | 211 | 448 | 194 | 159 | 353 | 162 | 148 | 310 |
| 15–19 | 384 | 328 | 712 | 336 | 312 | 648 | 279 | 279 | 558 | 281 | 255 | 536 | 226 | 217 | 443 | 258 | 218 | 476 |
| 20–24 | 535 | 492 | 1,027 | 522 | 480 | 1,002 | 490 | 421 | 911 | 458 | 455 | 913 | 440 | 439 | 879 | 452 | 399 | 851 |
| 25–29 | 708 | 603 | 1,311 | 717 | 570 | 1,287 | 710 | 552 | 1,262 | 756 | 605 | 1,361 | 709 | 586 | 1,295 | 676 | 618 | 1,294 |
| 30–34 | 935 | 754 | 1,689 | 802 | 665 | 1,467 | 732 | 652 | 1,384 | 826 | 672 | 1,498 | 761 | 622 | 1,383 | 835 | 652 | 1,487 |
| 35–39 | 1,196 | 960 | 2,156 | 1,174 | 845 | 2,019 | 1,148 | 826 | 1,974 | 1,012 | 794 | 1,806 | 1,022 | 859 | 1,881 | 946 | 757 | 1,703 |
| 40–44 | 1,338 | 973 | 2,311 | 1,245 | 867 | 2,112 | 1,175 | 864 | 2,039 | 1,154 | 849 | 2,003 | 1,201 | 928 | 2,129 | 1,163 | 913 | 2,076 |
| 45–49 | 1,489 | 1197 | 2,686 | 1,477 | 1123 | 2,600 | 1,426 | 1144 | 2,570 | 1,421 | 1032 | 2,453 | 1,311 | 1008 | 2,319 | 1,239 | 1004 | 2,243 |
| 50–54 | 1,473 | 1402 | 2,875 | 1,429 | 1368 | 2,797 | 1,400 | 1387 | 2,787 | 1,376 | 1244 | 2,620 | 1,437 | 1229 | 2,666 | 1,353 | 1265 | 2,618 |
| 55–59 | 1,638 | 1576 | 3,214 | 1,564 | 1488 | 3,052 | 1,564 | 1488 | 3,052 | 1,527 | 1329 | 2,856 | 1,394 | 1241 | 2,635 | 1,327 | 1215 | 2,542 |
| 60–64 | 1,203 | 1341 | 2,544 | 1,304 | 1327 | 2,631 | 1,330 | 1351 | 2,681 | 1,278 | 1226 | 2,504 | 1,377 | 1295 | 2,672 | 1,374 | 1213 | 2,587 |
| 65–69 | 809 | 1054 | 1,863 | 915 | 967 | 1,882 | 914 | 1014 | 1,928 | 872 | 971 | 1,843 | 950 | 1019 | 1,969 | 964 | 1024 | 1,988 |
| 70–74 | 599 | 907 | 1,506 | 627 | 827 | 1,454 | 651 | 783 | 1,434 | 667 | 796 | 1,463 | 642 | 747 | 1,389 | 660 | 730 | 1,390 |
| 75–79 | 486 | 722 | 1,208 | 518 | 730 | 1,248 | 455 | 722 | 1,177 | 443 | 638 | 1,081 | 403 | 553 | 956 | 423 | 541 | 964 |
| 80–84 | 203 | 411 | 614 | 247 | 398 | 645 | 227 | 411 | 638 | 245 | 387 | 632 | 225 | 399 | 624 | 220 | 383 | 603 |
| 85–89 | 51 | 170 | 221 | 83 | 157 | 240 | 95 | 194 | 289 | 79 | 154 | 233 | 76 | 155 | 231 | 100 | 159 | 259 |
| 90–94 | 17 | 38 | 55 | 13 | 44 | 57 | 12 | 42 | 54 | 9 | 39 | 48 | 21 | 34 | 55 | 19 | 53 | 72 |
| 95–99 | 3 | 11 | 14 | 5 | 8 | 13 | 2 | 14 | 16 | 1 | 5 | 6 | 2 | 7 | 9 | 3 | 15 | 18 |
| Total | 13,576 | 13,397 | 26,973 | 13,332 | 12,509 | 25,841 | 12,972 | 12,501 | 25,473 | 12,800 | 11,823 | 24,623 | 12,540 | 11,652 | 24,192 | 12,313 | 11,446 | 23,759 |

Abbreviations: M, males; F, females.

**Table S6.** Sensitivity analysis of baseline characteristics of individuals diagnosed with Bell’s palsy for each year.

|  | 2017 | 2018 | 2019 | 2020 | 2021 | 2022 | P^†^ |
| --- | --- | --- | --- | --- | --- | --- | --- |
|  | (N = 26,973) | (N = 25,841) | (N = 25,473) | (N = 24,623) | (N = 24,192) | (N = 23,759) |  |
| Age (yr) | * |  |  |  |  |  | <0.001 |
| 0–19 | 967 (3.59) | 687 (2.66) | 719 (2.82) | 767 (3.11) | 657 (2.72) | 588 (2.47) |  |
| 20–39 | 6895 (25.56) | 6423 (24.86) | 6089 (23.90) | 6114 (24.83) | 5881 (24.31) | 5811 (24.46) |  |
| 40–64 | 13630 (50.53) | 13192 (51.05) | 13129 (51.54) | 12436 (50.51) | 12421 (51.34) | 12066 (50.78) |  |
| ≥65 | 5481 (20.32) | 5539 (21.43) | 5536 (21.73) | 5306 (21.55) | 5233 (21.63) | 5294 (22.28) |  |
| Sex |  |  |  |  |  |  | <0.001 |
| Male | 13576 (50.33) | 13332 (51.59) | 12972 (50.92) | 12800 (51.98) | 12540 (51.84) | 12313 (51.82) |  |
| Female | 13397 (49.67) | 12509 (48.41) | 12501 (49.08) | 11823 (48.02) | 11652 (48.16) | 11446 (48.18) |  |
| Type of insurance |  |  |  |  |  |  | 0.678 |
| NHI | 26002 (96.40) | 24920 (96.44) | 24584 (96.51) | 23762 (96.50) | 23386 (96.67) | 22926 (96.49) |  |
| Medical aid | 971 (3.60) | 921 (3.56) | 889 (3.49) | 861 (3.50) | 806 (3.33) | 833 (3.51) |  |
| Systemic diseases |  |  |  |  |  |  |  |
| Hypertension | 7115 (26.38)* | 7050 (27.28) | 6976 (27.39) | 6599 (26.80) | 6570 (27.16) | 6485 (27.29) | 0.007 |
| Diabetes | 4764 (17.66)* | 4917 (19.03) | 4808 (18.87) | 4770 (19.37) | 4815 (19.90)* | 4878 (20.53)* | <0.001 |
| Dyslipidemia | 6751 (25.03)* | 7451 (28.83)* | 8079 (31.72) | 8166 (33.16)* | 8635 (35.69)* | 9093 (38.27)* | <0.001 |
| Chronic kidney disease | 254 (0.94) | 231 (0.89) | 276 (1.08) | 243 (0.99) | 258 (1.07) | 252 (1.06) | 0.179 |
| Malignancy | 3025 (11.21)* | 2732 (10.57) | 2590 (10.17) | 2331 (9.47)* | 2213 (9.15)* | 2087 (8.78)* | <0.001 |
| Hyperthyroidism | 350 (1.30)* | 391 (1.51) | 426 (1.67) | 447 (1.82) | 490 (2.03)* | 488 (2.05)* | <0.001 |
| Hypothyroidism | 841 (3.12)* | 925 (3.58)* | 1104 (4.33) | 1054 (4.28) | 1158 (4.79) | 1257 (5.29)* | <0.001 |
| Chronic liver disease | 1439 (5.33)* | 1683 (6.51)* | 1968 (7.73) | 1995 (8.10) | 2103 (8.69)* | 2249 (9.47)* | <0.001 |
| Autoimmune diseases | 1786 (6.62)* | 1968 (7.62) | 2084 (8.18) | 2258 (9.17)* | 2430 (10.04)* | 2519 (10.60)* | <0.001 |

Data are presented as N (%)

^†^P-value from ANOVA; <0.05 was considered statistically significant.

^*^P<0.01 by post-hoc Bonferroni test vs. 2019.

Abbreviations: NHI, national health insurance; CKD, chronic kidney disease.

**Supplementary Table S7.** Sensitivity analysis of the comparisons of incidence rate ratio of Bell’s palsy between pre- and post-COVID-19.

|  | Year | Residents | Events | Crude incidence rate (per 100,000 persons) |  | Unadjusted IRR  (95% CI) | P |  | Age, sex-adjusted IRR (95% CI) | P |
| --- | --- | --- | --- | --- | --- | --- | --- | --- | --- | --- |
| Total | 2017 | 51,226,047.0 | 26,973 | 52.65 |  | 1.06 (1.04–1.08) | <0.001 |  | 1.08 (1.06–1.10) | <0.001 |
|  | 2018 | 51,295,997.0 | 25,841 | 50.38 |  | 1.02 (1.00–1.03) | 0.088 |  | 1.03 (1.01–1.04) | 0.005 |
|  | 2019 | 51,332,197.0 | 25,473 | 49.62 |  | 1 (reference) |  |  | 1 (reference) |  |
|  | 2020 | 51,343,421.5 | 24,623 | 47.96 |  | 0.97 (0.95–0.98) | <0.001 |  | 0.96 (0.94–0.97) | <0.001 |
|  | 2021 | 51,326,671.0 | 24,192 | 47.13 |  | 0.95 (0.93–0.97) | <0.001 |  | 0.93 (0.92–0.95) | <0.001 |
|  | 2022 | 51,251,980.5 | 23,759 | 46.36 |  | 0.93 (0.92–0.95) | <0.001 |  | 0.91 (0.89–0.93) | <0.001 |
| Males | 2017 | 25,576,145.0 | 13,576 | 53.08 |  | 1.05 (1.02–1.07) | <0.001 |  | 1.07 (1.04–1.09) | <0.001 |
|  | 2018 | 25,601,323.0 | 13,332 | 52.08 |  | 1.03 (1.00–1.05) | 0.025 |  | 1.04 (1.01–1.06) | 0.003 |
|  | 2019 | 25,608,606.5 | 12,972 | 50.65 |  | 1 (reference) |  |  | 1 (reference) |  |
|  | 2020 | 25,605,186.0 | 12,800 | 49.99 |  | 0.99 (0.96–1.01) | 0.2890 |  | 0.98 (0.95–1.00) | 0.074 |
|  | 2021 | 25,588,082.0 | 12,540 | 49.01 |  | 0.97 (0.94–0.99) | 0.008 |  | 0.95 (0.93–0.97) | <0.001 |
|  | 2022 | 25,538,938.0 | 12,313 | 48.21 |  | 0.95 (0.93–0.98) | <0.001 |  | 0.93 (0.9–0.95) | <0.001 |
| Females | 2017 | 25,649,902.0 | 13,397 | 52.23 |  | 1.07 (1.05–1.10) | <0.001 |  | 1.10 (1.07–1.12) | <0.001 |
|  | 2018 | 25,694,674.0 | 12,509 | 48.68 |  | 1.00 (0.98–1.03) | 0.889 |  | 1.01 (0.99–1.04) | 0.353 |
|  | 2019 | 25,723,590.5 | 12,501 | 48.60 |  | 1 (reference) |  |  | 1 (reference) |  |
|  | 2020 | 25,738,235.5 | 11,823 | 45.94 |  | 0.95 (0.92–0.97) | <0.001 |  | 0.94 (0.91–0.96) | <0.001 |
|  | 2021 | 25,738,589.0 | 11,652 | 45.27 |  | 0.93 (0.91–0.96) | <0.001 |  | 0.91 (0.89–0.94) | <0.001 |
|  | 2022 | 25,713,042.5 | 11,446 | 44.51 |  | 0.92 (0.89–0.94) | <0.001 |  | 0.89 (0.87–0.91) | <0.001 |

^*^IRR=annual mean incidence of Bell’s palsy during the year/annual mean incidence in 2019.

P<0.05 was considered statistically significant.

Abbreviations: IRR, incidence rate ratio; CI, confidence interval.

**Supplementary Table S8.** Sensitivity analysis of the comparisons of incidence rate ratio of Bell’s palsy between pre- and post-COVID-19 by age groups.

| Age group | Year | Residents | Events | Crude incidence rate (per 100,000 persons) |  | Unadjusted IRR  (95% CI) | P |  | Age, sex-adjusted IRR (95% CI) | P |
| --- | --- | --- | --- | --- | --- | --- | --- | --- | --- | --- |
| 0–19 | 2017 | 9878328.5 | 1679 | 17.00 |  | 1.23 (1.15–1.33) | <0.001 |  | 1.22 (1.13–1.31) | <0.001 |
|  | 2018 | 9572709.5 | 1335 | 13.95 |  | 1.01 (0.94–1.09) | 0.761 |  | 1.01 (0.93–1.09) | 0.856 |
|  | 2019 | 9266584.0 | 1277 | 13.78 |  | 1 (reference) |  |  | 1 (reference) |  |
|  | 2020 | 8932022.5 | 1303 | 14.59 |  | 1.06 (0.98–1.14) | 0.148 |  | 1.06 (0.98–1.15) | 0.126 |
|  | 2021 | 8605344.0 | 1100 | 12.78 |  | 0.93 (0.86–1.01) | 0.068 |  | 0.93 (0.86–1.01) | 0.098 |
|  | 2022 | 8343315.5 | 1064 | 12.75 |  | 0.93 (0.85–1.00) | 0.062 |  | 0.93 (0.86–1.01) | 0.087 |
| 20–39 | 2017 | 14139875.5 | 6183 | 43.73 |  | 1.10 (1.06–1.14) | <0.001 |  | 1.10 (1.06–1.14) | <0.001 |
|  | 2018 | 14042917.5 | 5775 | 41.12 |  | 1.03 (1.00–1.07) | 0.083 |  | 1.03 (1.00–1.07) | 0.086 |
|  | 2019 | 13896166.0 | 5531 | 39.80 |  | 1 (reference) |  |  | 1 (reference) |  |
|  | 2020 | 13694461.0 | 5578 | 40.73 |  | 1.02 (0.99–1.06) | 0.224 |  | 1.02 (0.99–1.06) | 0.200 |
|  | 2021 | 13458179.0 | 5438 | 40.41 |  | 1.02 (0.98–1.05) | 0.430 |  | 1.02 (0.98–1.06) | 0.327 |
|  | 2022 | 13152595.0 | 5335 | 40.56 |  | 1.02 (0.98–1.06) | 0.324 |  | 1.02 (0.98–1.06) | 0.271 |
| 40–64 | 2017 | 20146299.0 | 13630 | 67.66 |  | 1.05 (1.03–1.08) | <0.001 |  | 1.06 (1.04–1.09) | <0.001 |
|  | 2018 | 20295779.0 | 13192 | 65.00 |  | 1.01 (0.99–1.04) | 0.305 |  | 1.02 (0.99–1.04) | 0.197 |
|  | 2019 | 20456058.0 | 13129 | 64.18 |  | 1 (reference) |  |  | 1 (reference) |  |
|  | 2020 | 20588101.0 | 12436 | 60.40 |  | 0.94 (0.92–0.96) | <0.001 |  | 0.94 (0.92–0.96) | <0.001 |
|  | 2021 | 20691899.5 | 12421 | 60.03 |  | 0.94 (0.91–0.96) | <0.001 |  | 0.93 (0.91–0.96) | <0.001 |
|  | 2022 | 20752695.0 | 12066 | 58.14 |  | 0.91 (0.88–0.93) | <0.001 |  | 0.90 (0.88–0.93) | <0.001 |
| 65–99 | 2017 | 7061544.0 | 5481 | 77.62 |  | 1.08 (1.04–1.12) | <0.001 |  | 1.08 (1.04–1.12) | <0.001 |
|  | 2018 | 7384591.0 | 5539 | 75.01 |  | 1.05 (1.01–1.08) | 0.020 |  | 1.04 (1.00–1.08) | 0.027 |
|  | 2019 | 7713389.0 | 5536 | 71.77 |  | 1 (reference) |  |  | 1 (reference) |  |
|  | 2020 | 8128837.0 | 5306 | 65.27 |  | 0.91 (0.88–0.94) | <0.001 |  | 0.91 (0.88–0.95) | <0.001 |
|  | 2021 | 8571248.5 | 5233 | 61.05 |  | 0.85 (0.82–0.88) | <0.001 |  | 0.85 (0.82–0.88) | <0.001 |
|  | 2022 | 9003375.0 | 5294 | 58.80 |  | 0.82 (0.79–0.85) | <0.001 |  | 0.82 (0.79–0.85) | <0.001 |

^*^IRR=annual mean incidence of ON during the year/annual mean incidence in 2019.

P<0.05 was considered statistically significant.

Abbreviations: IRR, incidence rate ratio; CI, confidence interval.


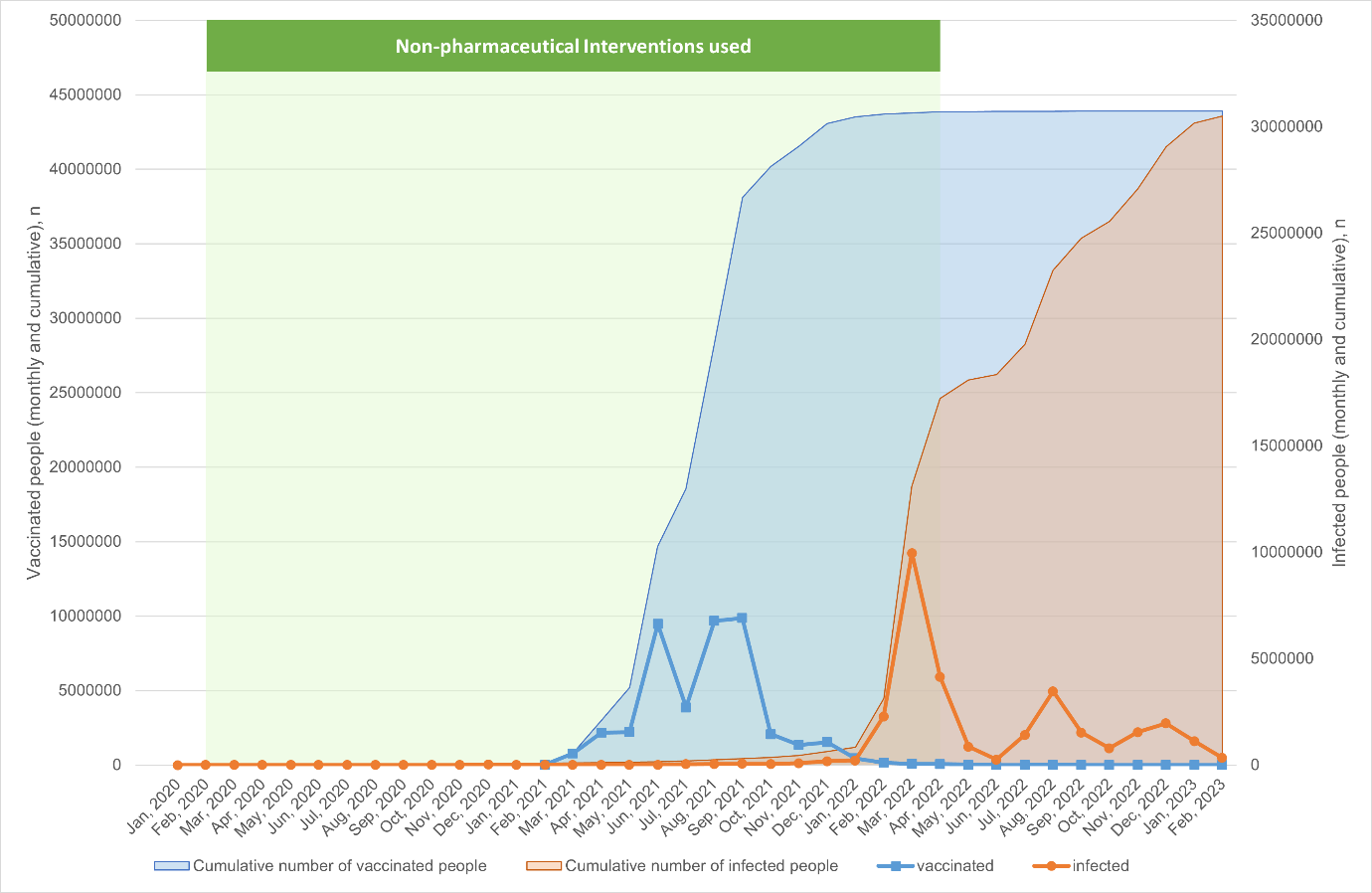


**Supplementary Figure S1. Trends in cumulative SARS-CoV-2 vaccination and infection in Korea between 2020 and 2023.** The thick blue line with square dots represents the monthly number of vaccinated individuals while the thick orange line with circle dots represents the monthly number of individuals with COVID-19 in South Korea from January 2020 to February 2023. The green shaded area indicates the period during which non-pharmaceutical interventions (NPIs) were enforced nationwide, starting in February 2020. A nationwide vaccination program began in February 2021, leading to a rapid increase in vaccination coverage. SARS-CoV-2 infections surged dramatically in March 2022 (orange shaded area). The blue shaded area represents the cumulative number of vaccinated people, and the orange shaded area represents the cumulative number of people with COVID-19. Abbreviations: BP, Bell’s palsy; SARS-CoV-2, Severe acute respiratory syndrome coronavirus 2; COVID-19, coronavirus disease-19.


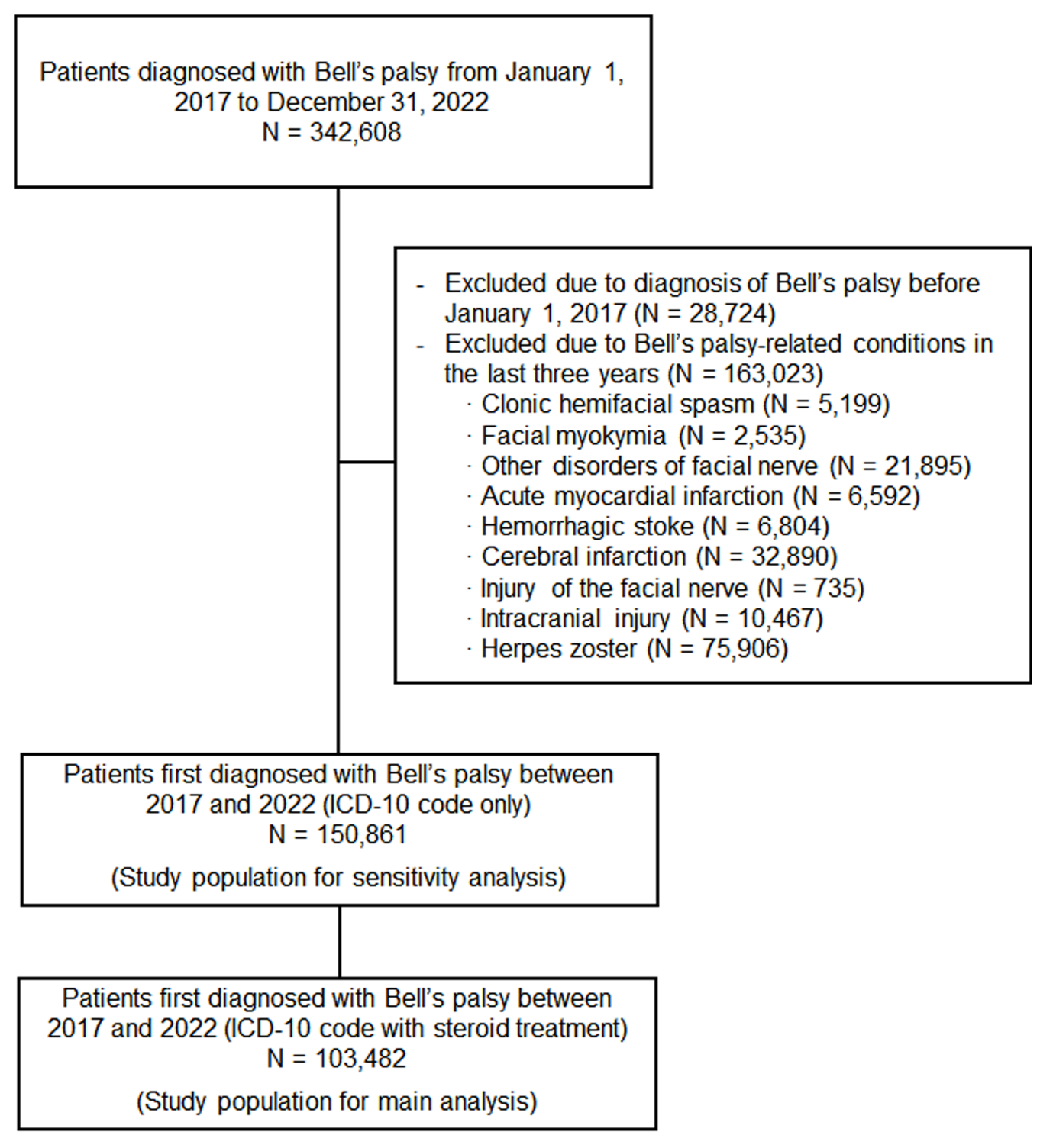


**Supplementary Figure S2. Flowchart of study population selection.** From 342,608 individuals with BP diagnosis between January 2017 and December 2022, those diagnosed with BP before 1 January 2017 (N=28,724) were excluded. Those diagnosed with BP-related conditions (N=163,023) were excluded, resulting in a total of 150,861 individuals. Among them, 103,482 individuals who were prescribed glucocorticoid treatment within 60 days of their BP diagnosis were included in the main analysis. Abbreviations: BP, Bell’s palsy.

**
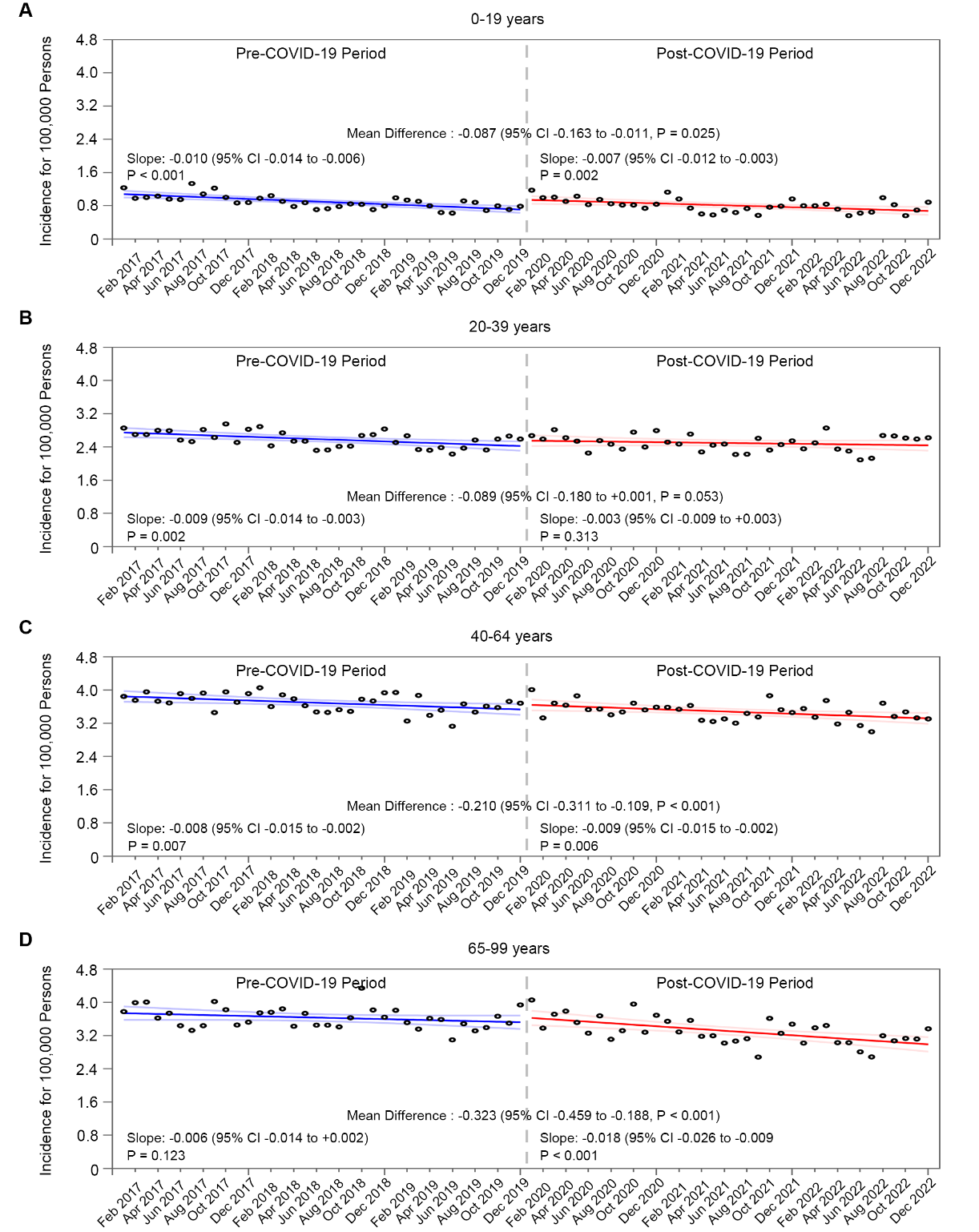
**

**Supplementary Figure S3. Trends in monthly incidence of BP before and after the COVID-19 outbreak by age groups.** The bold slope lines were estimated using the segmented regression model for (A) individuals aged 0–19 years, (B) individuals aged 20–39 years, (C) individuals aged 40–64 years, and (D) individuals aged 65–99 years. The bold blue line indicates the pre-COVID-19 period from 2017 to 2019, whereas the red bold line denotes the beginning of non-pharmaceutical interventions for COVID-19. The light blue and light red lines represent the 95% CI for the pre-COVID-19 and COVID-19 periods, respectively. Abbreviations: BP, Bell’s palsy; COVID-19, coronavirus disease-19; CI, confidence interval.


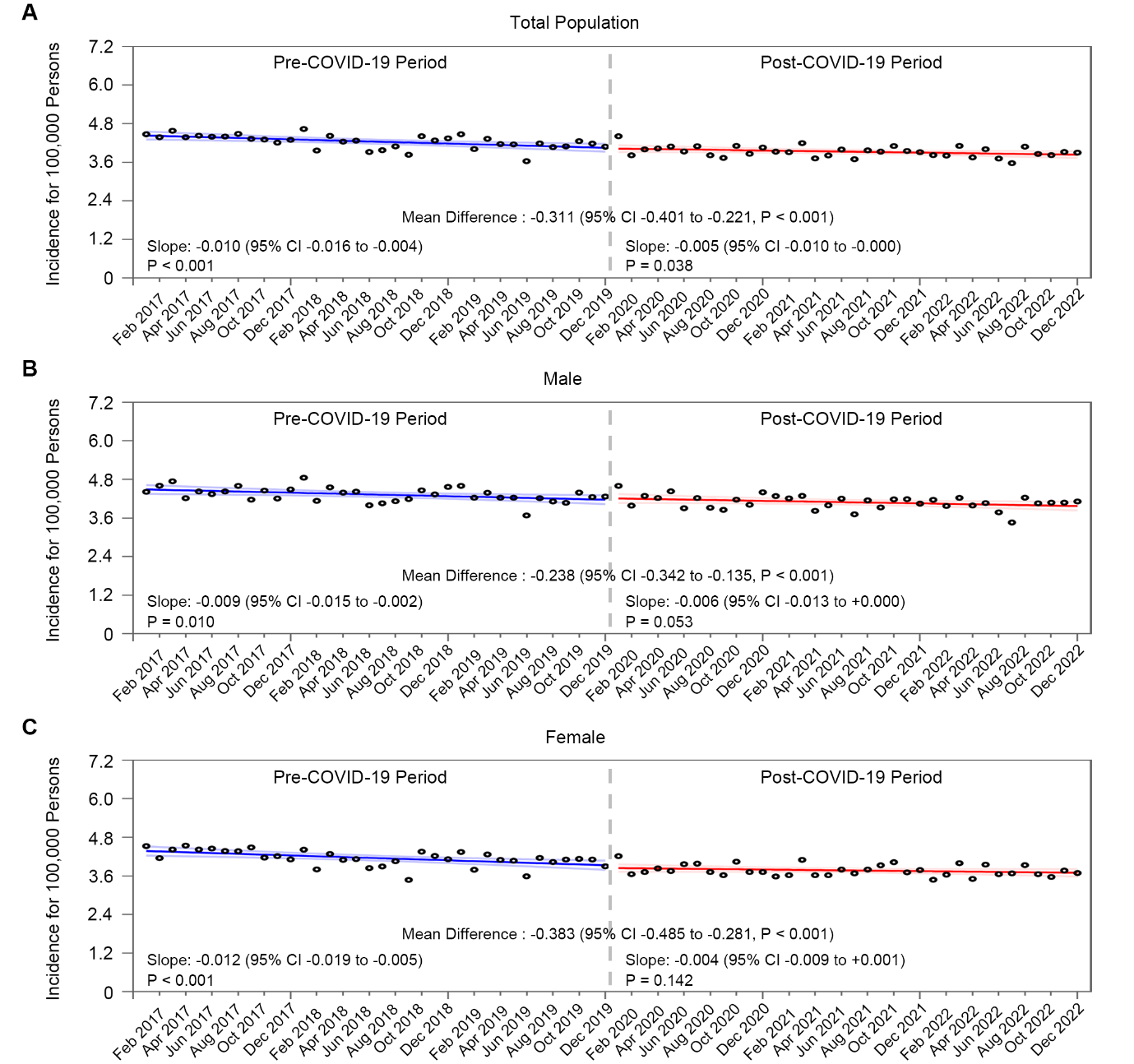


**Supplementary Figure S4. Sensitivity analysis of trends in monthly BP incidence before and after the COVID-19 outbreak by sex.** The bold slope lines were estimated using the segmented regression model for (A) Total population, (B) Males, and (C) Females. The bold blue line indicates the pre-COVID-19 period from 2017 to 2019, whereas the red bold line denotes the beginning of non-pharmaceutical interventions for COVID-19. The light blue and light red lines represent the 95% CI for the pre-COVID-19 and COVID-19 periods, respectively. Abbreviations: BP, Bell’s palsy; COVID-19, coronavirus disease-19; CI, confidence interval.


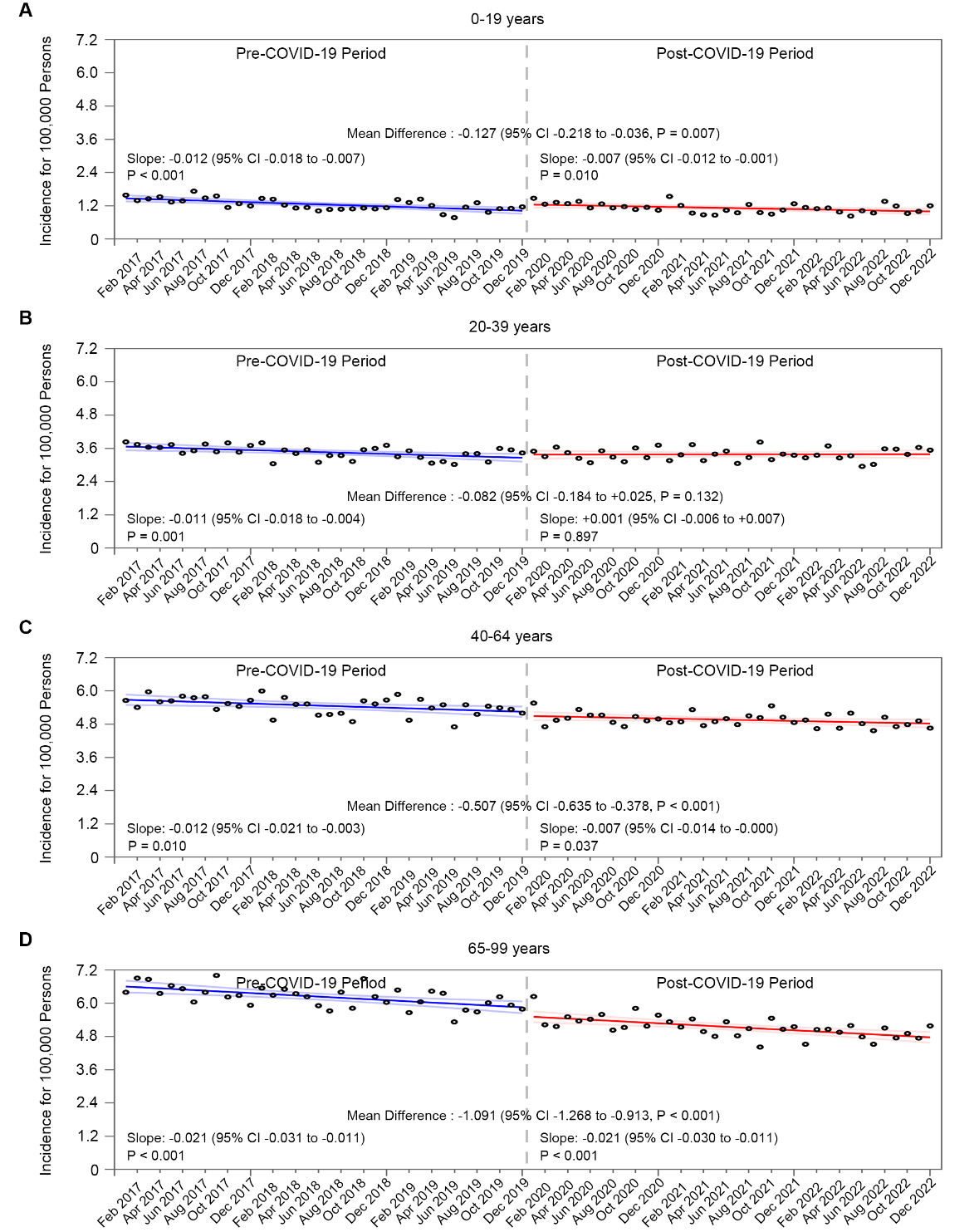


**Supplementary Figure S5. Sensitivity analysis of trends in monthly BP incidence before and after the COVID-19 outbreak by age groups.** The bold slope lines were estimated using the segmented regression model for (A) individuals aged 0–19 years, (B) individuals aged 20–39 years, (C) individuals aged 40–64 years, and (D) individuals aged 65–99 years. The bold blue line indicates the pre-COVID-19 period from 2017 to 2019, whereas the red bold line denotes the beginning of non-pharmaceutical interventions for COVID-19. The light blue and light red lines represent the 95% CI for the pre-COVID-19 and COVID-19 periods, respectively. Abbreviations: BP, Bell’s palsy; COVID-19, coronavirus disease-19; CI, confidence interval.


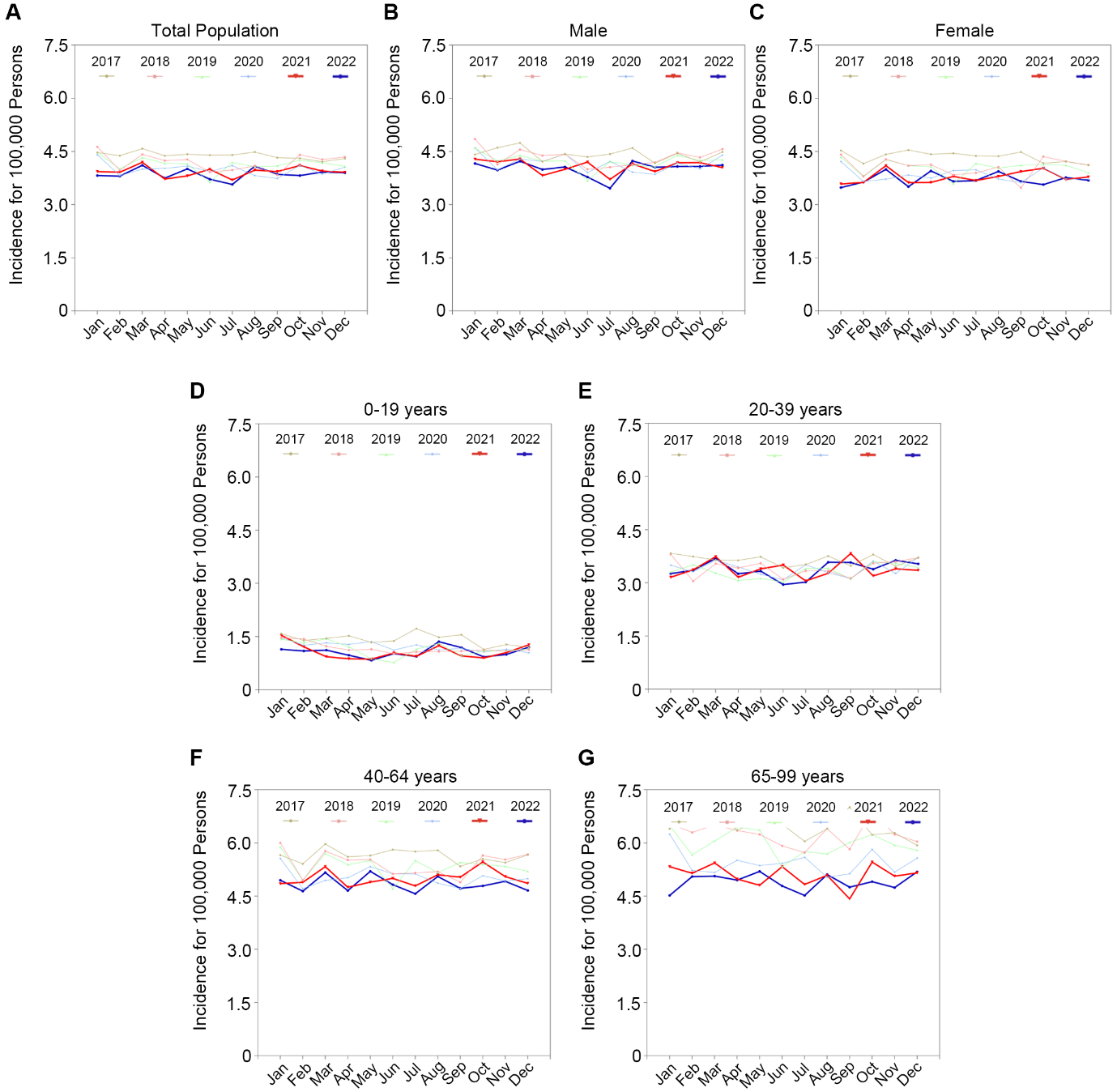


**Supplementary Figure S6.** **Sensitivity analysis of monthly BP incidence from 2017 to 2022.** The thick red line indicates the observed monthly BP incidence in 2021, while the thick blue line represents the observed BP incidence in 2022 for the following groups: (A) total population, (B) males, (C) females, (D) individuals aged 0–19 years, (E) individuals aged 20–39 years, (F) individuals aged 40–64 years, and (G) individuals aged 65–99 years. Abbreviation: BP, Bell’s palsy


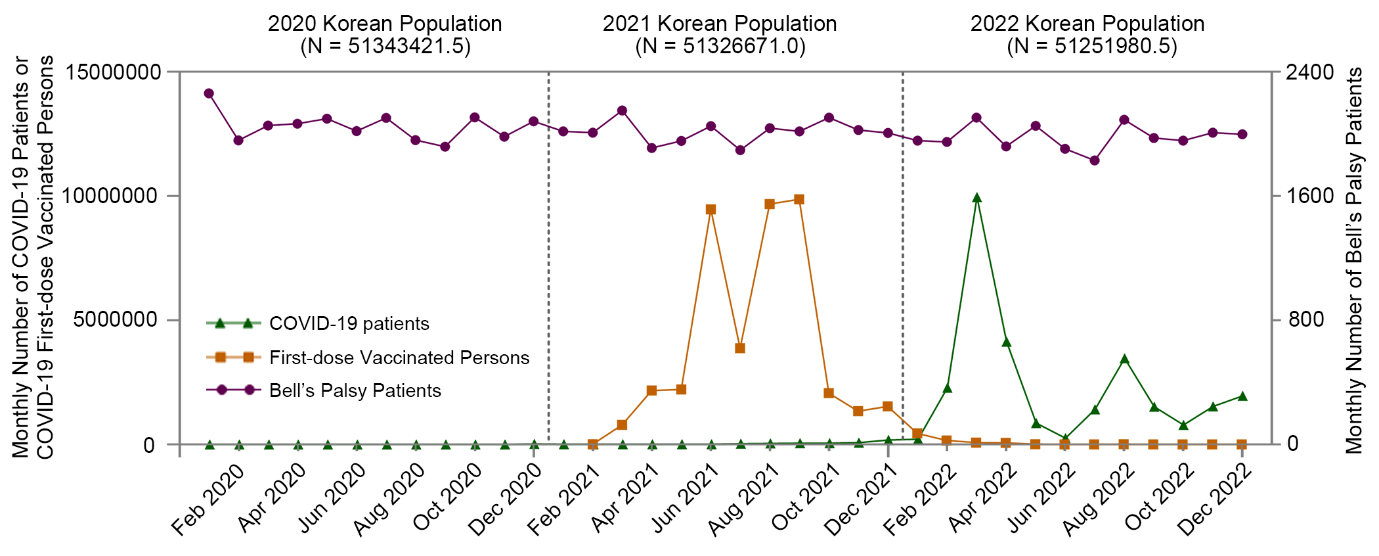


**Supplementary Figure S7.** **Sensitivity analysis of the changes in monthly number of infections, vaccinations, and BP between 2020 and 2022.** The purple line with circle dots denotes the monthly number of patients with BP; the orange line with square dots denotes the number of vaccinated persons; and the green line with triangle dots denotes the number of SARS-CoV-2 infections. Abbreviations: BP, Bell’s palsy; SARS-CoV-2, severe acute respiratory syndrome coronavirus 2.
